# Dual-Model Artificial Intelligence Framework Integrating AI-Based Markerless Motion Capture for Dynamic Gait Prediction and Health-Status Classification

**DOI:** 10.3390/diagnostics16162588

**Published:** 2026-08-16

**Authors:** Edder Jair Rodríguez-Granados, Guillermo Urriolagoitia-Sosa, Beatriz Romero-Ángeles, Jorge Alberto Gomez-Niebla, Jonathan Rodolfo Guereca-Ibarra, Maria de la Luz Suarez-Hernandez, Manuel Nazario Rocha-Martinez, Eduardo Enrique Carmona-Hernández, Luis Itzcoatl Lugo-Chacon, Gabriela Ramirez-Sanchez

**Affiliations:** Sección de Estudios de Posgrado e Investigación, Escuela Superior de Ingeniería Mecánica y Eléctrica, Unidad Profesional Adolfo López Mateos Zacatenco, Instituto Politécnico Nacional, Edificio 5, 2do. Piso, Col. Lindavista, Alc. Gustavo A. Madero, Ciudad de México 07300, Mexico; jgomezn2200@alumno.ipn.mx (J.A.G.-N.); jguerecai2200@alumno.ipn.mx (J.R.G.-I.); luzsuarez398@gmail.com (M.d.l.L.S.-H.); mrocham2201@alumno.ipn.mx (M.N.R.-M.); ecarmonah2300@alumno.ipn.mx (E.E.C.-H.); llugoc1700@alumno.ipn.mx (L.I.L.-C.); gramirezs2500@alumno.ipn.mx (G.R.-S.)

**Keywords:** artificial intelligence, gait analysis, markerless motion capture, ground reaction forces, center of pressure, deep learning, LSTM, biomechanics, dynamic prediction, clinical decision support

## Abstract

**Background/Objectives**: Human gait analysis is essential for identifying biomechanical alterations associated with pathological conditions. However, conventional laboratory systems that combine optical motion capture and force plates remain costly, space-demanding, and difficult to implement in routine or accessible settings. This study proposes a dual-model artificial intelligence framework designed to bridge kinematics and dynamics and subsequently support gait health-status classification from motion data. **Methods**: The first model was developed to estimate ground reaction forces (GRF) and center of pressure (CoP) signals from kinematic inputs. Public datasets containing synchronized kinematics and dynamics were used to train and evaluate long short-term memory (LSTM) and one-dimensional convolutional neural network (CNN1D) architectures. A robustness stage further adapted the dynamic prediction model to markerless-like kinematic inputs through domain-adaptation training. The second model was implemented as a multichannel convolutional classifier using normalized GRF/CoP signals and metadata. Three variants were compared: signals only, signals with minimal metadata, and signals with rich metadata. Finally, a bridge block connected both models, and a proof-of-concept deployment was performed using markerless kinematics obtained with Move AI and Blender. **Results**: The final dynamic prediction model achieved an overall RMSE of 0.0676, with reconstructed-signal RMSE of 0.0500 and reconstructed contact accuracy of 0.9736. The best classification variant achieved a balanced accuracy of 0.9608, while the minimal-metadata variant was selected for pipeline integration due to its compatibility with accessible data acquisition. In the full pipeline evaluation, the predicted dynamics correctly classified the healthy-control samples. In the Move AI/Blender proof-of-concept, all five healthy participants were classified as healthy controls, with a mean non-pathological classification probability consistent with this outcome. **Conclusions**: The proposed dual-model framework demonstrates the operational feasibility of linking kinematic acquisition, dynamic prediction, and gait classification within a single artificial intelligence pipeline. The Move AI/Blender stage represents a preliminary proof of concept rather than clinical validation, and further evaluation with pathological participants and synchronized force-plate measurements is required.

## 1. Introduction

Human gait is a complex motor function that reflects the interaction between the nervous, musculoskeletal, and biomechanical systems. Alterations in gait may reveal functional limitations, compensatory mechanisms, or pathological conditions; therefore, quantitative gait analysis has become an important tool for clinical assessment, rehabilitation monitoring, treatment planning, and biomechanical research [1,2,3]. In clinical contexts, instrumented gait analysis can provide objective information that complements observational assessment and supports clinical decision-making, particularly in populations with neurological or musculoskeletal disorders [2,3]. However, despite its value, objective gait assessment is still not universally available in routine practice, mainly because the most complete measurement systems require specialized infrastructure, technical expertise, and controlled acquisition conditions [1,4].

Conventional laboratory-based gait analysis commonly relies on marker-based optical motion capture synchronized with force plates. These systems remain reference methods for obtaining three-dimensional kinematics and kinetic variables because they provide high-resolution measurements of body motion and external loading [3,4,5]. Among the kinetic variables used in gait analysis, ground reaction forces (GRF) and center of pressure (CoP) are particularly relevant because they describe the interaction between the body and the ground during stance. GRF components provide information about vertical loading, braking, propulsion, and mediolateral control, whereas CoP trajectories reflect the progression of plantar loading and balance control during foot–ground contact [6,7]. Nevertheless, laboratory systems are limited by high cost, preparation time, marker placement sensitivity, soft-tissue artifacts, the need for trained operators, and reduced ecological validity outside controlled environments [4,8,9]. In addition, force plates require clean foot contact with the instrumented areas, which can reduce the number of usable trials and complicate data acquisition in pathological populations.

Recent advances in markerless motion capture, computer vision, and artificial intelligence (AI) have created new opportunities for more accessible gait assessment. Markerless systems can estimate body motion from video without reflective markers, reducing preparation time and enabling data collection in more natural and scalable environments [8,9]. Systematic reviews and validation studies have reported promising agreement between markerless and marker-based systems for several gait-related variables, especially spatiotemporal parameters and sagittal-plane lower-limb kinematics [5,8,9,10,11,12]. However, markerless motion capture is not yet fully interchangeable with marker-based laboratory systems, and its accuracy may depend on the anatomical region, plane of motion, camera configuration, task, processing pipeline, and population studied [8,10,11,12,13]. These findings suggest that markerless kinematics should be used carefully, particularly when clinical or dynamic interpretation is required.

At the same time, machine learning and deep learning methods have become increasingly relevant for biomechanical prediction and gait classification. Large public datasets such as GroundLink provide synchronized kinematics, GRF, and CoP, enabling data-driven models to learn relationships between body movement and ground reaction dynamics [7]. Similarly, the Camargo dataset, accessed through AddBiomechanics in this study, provides lower-limb motion and ground reaction force data across multiple locomotion modes, while AddBiomechanics offers a standardized processing framework and large-scale resource for developing models that connect motion and dynamics [14,15]. For classification tasks, GaitRec has shown the value of large-scale GRF datasets for studying healthy and impaired gait, while deep learning models such as GaitRec-Net have demonstrated that GRF patterns can distinguish healthy controls from gait-disorder cases [16,17]. More recent work has also explored the estimation of GRF and CoP from markerless motion capture or other motion-derived inputs, supporting the feasibility of approximating dynamic variables that would otherwise require force plates [12,13,18,19].

Despite this progress, many existing approaches address only one part of the problem. Some studies focus on estimating kinetic variables from motion data, whereas others classify gait patterns using already measured dynamic signals. A system that only classifies gait from measured GRF and CoP still depends on force plates or equivalent kinetic sensors; conversely, a model that estimates GRF and CoP from kinematics does not automatically demonstrate whether the estimated dynamics remain useful for downstream classification. Therefore, an important methodological gap remains: the lack of an integrated framework that connects accessible kinematic acquisition, dynamic prediction, and gait health-status classification within a single operational workflow.

To address this gap, this study proposes a dual-model AI framework for dynamic gait prediction and health-status classification. The first model estimates bilateral GRF and CoP signals from kinematic inputs, producing ten dynamic channels corresponding to vertical, anteroposterior, and mediolateral GRF components, together with anteroposterior and mediolateral CoP trajectories for both feet. The second model classifies gait health status using normalized dynamic signals and subject metadata. Between both models, a bridge block reconstructs continuous dynamic predictions, extracts support phases, processes CoP signals, normalizes each sample to 101 points, and generates a standardized 101 × 10 input compatible with the classifier. In addition, a robustness stage adapts the dynamic prediction model to markerless-like kinematic inputs, and a proof-of-concept deployment is performed using markerless kinematics obtained with Move AI and Blender.

The main contributions of this work are fourfold. First, a kinematics-to-dynamics model is developed and validated for estimating GRF and CoP from motion data. Second, a dynamics-to-classification model is evaluated through three input variants, including a final configuration based on minimal metadata to support practical deployment. Third, a bridge block is implemented to connect both models into an end-to-end workflow. Finally, the complete framework is tested in a preliminary proof-of-concept deployment using markerless motion-capture data to assess its operational feasibility with accessible kinematic inputs. This stage does not constitute clinical validation and requires further evaluation with pathological participants and synchronized force-plate measurements.

## 2. Materials and Methods

### 2.1. Dual-Model Pipeline and Overall Study Design

This study proposes a staged artificial intelligence framework for gait analysis, designed to transform kinematic information into predicted dynamic variables and subsequently use those variables for gait health-status classification. The framework combines two artificial intelligence models connected through an intermediate processing stage and was developed and evaluated progressively through five experiments.

The dual-model pipeline consists of a kinematics-to-dynamics prediction model, referred to as Model 1, and a dynamics-to-classification model, referred to as Model 2. Model 1 receives lower-limb kinematic information and estimates bilateral ground reaction forces (GRF) and center of pressure (CoP) trajectories. Its output comprises ten dynamic channels: vertical, anteroposterior, and mediolateral GRF components, together with anteroposterior and mediolateral CoP coordinates for the left and right feet. Auxiliary left- and right-foot contact outputs were also incorporated to improve the temporal consistency of the predicted support phases. The dynamic prediction stage was first trained and validated using synchronized kinematic and kinetic datasets, and later adapted to markerless-like kinematic representations to evaluate its robustness under conditions closer to accessible motion capture systems.

Model 2 was designed as a gait classification model using normalized dynamic signals as input. The classifier receives a fixed 101 × 10 representation, where each sample contains the ten dynamic channels normalized over the support phase. Three input configurations were evaluated: dynamic signals alone, dynamic signals with minimal metadata, and dynamic signals with rich metadata. For integration into the complete pipeline, the version with minimal metadata was selected because it requires only variables that can realistically be obtained in an accessible acquisition scenario: sex, height, and body weight.

A bridge block was implemented to connect Model 1 and Model 2. This block reconstructs continuous predicted dynamic signals from overlapping windows, extracts support-phase segments, applies biomechanical processing to the CoP, normalizes each segment to 101 time points, and arranges the final dynamic representation in the channel order required by the classifier. This intermediate stage is essential because Model 1 produces continuous trial-level dynamic predictions, whereas Model 2 requires standardized support-phase samples. At the system level, the resulting data flow is as follows: kinematic inputs → Model 1 → predicted GRF/CoP → bridge block → standardized 101 × 10 dynamic representation → Model 2 → gait classification.

**Figure 1:**
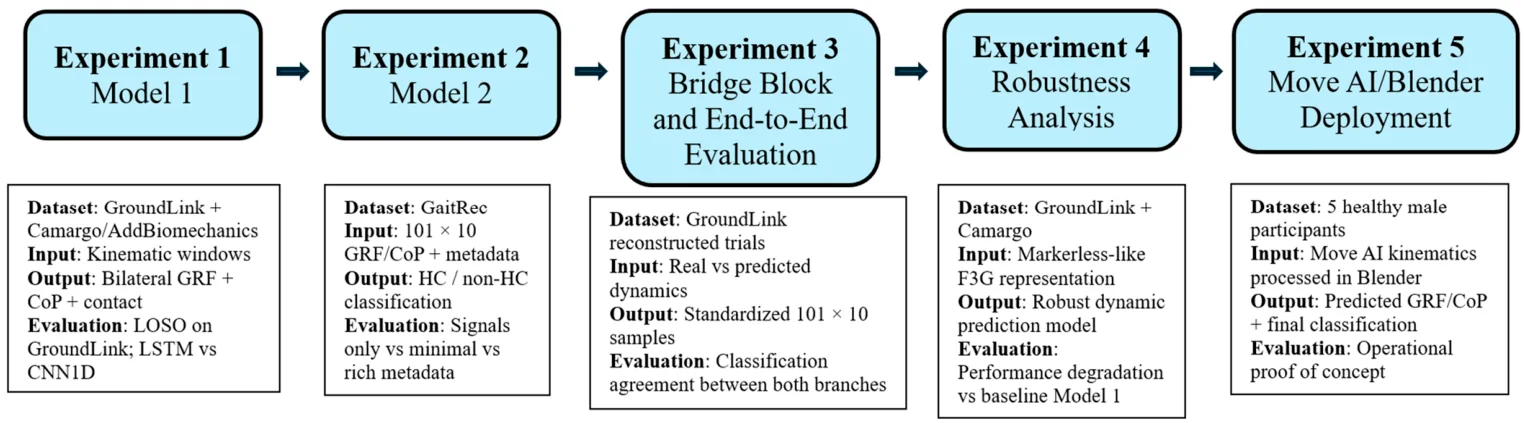
Comprehensive overview of the five experimental stages of the proposed dual-model framework. Experiment 1 develops and validates the kinematics-to-dynamics model (Model 1) using GroundLink and Camargo/AddBiomechanics data. Experiment 2 develops and compares the dynamics-to-classification model (Model 2) using GaitRec data. Experiment 3 integrates both models through the bridge block and evaluates the end-to-end pipeline using measured and predicted dynamics. Experiment 4 assesses the robustness of Model 1 under markerless-like input conditions using the F3G representation. Experiment 5 demonstrates the operational feasibility of the complete framework through a proof-of-concept deployment using Move AI and Blender data.

At the study-design level, the proposed framework was developed and evaluated through five sequential experiments, as summarized in Figure 1. Experiment 1 developed and validated the kinematics-to-dynamics prediction model (Model 1) using synchronized kinematic and kinetic data. Experiment 2 developed and compared three variants of the dynamics-to-classification model (Model 2). Experiment 3 integrated both models through the bridge block and evaluated the end-to-end pipeline by comparing classification outcomes obtained from measured and predicted dynamics. Experiment 4 assessed the robustness of Model 1 under markerless-like input conditions using the F3G representation. Finally, Experiment 5 implemented a proof-of-concept deployment of the complete framework using markerless kinematic data obtained with Move AI (Move AI Limited, London, UK; https://www.move.ai/, accessed on 14 August 2026) and processed in Blender 4.3 (Blender Foundation, Amsterdam, The Netherlands). Together, these experiments progressively evaluated the individual models, their integration, their robustness to markerless-like inputs, and their final operational deployment. Figure 1 summarizes the datasets, inputs, outputs, and evaluation objectives associated with the five experimental stages.

### 2.2. Datasets and Experimental Data Sources

Several data sources were used in this study according to the specific requirements of each stage of the proposed framework. The dynamic prediction model required datasets containing synchronized kinematic and kinetic information, whereas the classification model required a large-scale dataset of standardized dynamic gait signals with health-status labels. Finally, markerless motion-capture data were acquired and processed to test the complete workflow under accessible deployment conditions. Therefore, each data source was incorporated with a specific methodological purpose.

GroundLink dataset. GroundLink was used as the primary reference dataset for developing and evaluating Model 1. This dataset contains synchronized full-body motion-capture data and ground reaction dynamics, including ground reaction forces (GRF) and center of pressure (CoP). It includes data from seven participants, comprising 368 processed motion trials, approximately 1.59 million recorded frames, and 19 different movements [7]. The acquisition system used high-resolution optical motion capture and embedded force platforms, with a marker set of 96 markers and synchronized force measurements [7]. In the present study, walking trials from GroundLink were used to construct supervised samples, with kinematic variables as model inputs and bilateral GRF/CoP signals as dynamic targets. Of the seven participants described in the original dataset, six were included in the present Model 1 analysis. Subject s007 was excluded because the data available for this study did not contain the corresponding C3D marker-trajectory files required to construct the kinematic inputs, although kinetic data files were available. Therefore, the final subject-wise evaluation comprised subjects s001–s006. Because these data provide synchronized kinematics and measured dynamics, GroundLink served as the primary reference for training, validating, reconstructing, and testing the kinematics-to-dynamics prediction stage.

Camargo/AddBiomechanics data. The Camargo data, accessed through AddBiomechanics (Stanford University, Stanford, CA, USA; https://addbiomechanics.org/, accessed on 14 August 2026), were incorporated as an additional source of biomechanical locomotion information during the development of Model 1 and later during the robustness analysis. The Camargo dataset contains data from 22 able-bodied adults performing multiple locomotion modes, including level-ground walking, treadmill walking, ramp ascent/descent, and stair ascent/descent [14]. The dataset includes motion-capture data, ground reaction forces, wearable sensor signals, and processed joint-level biomechanical variables [14,15]. The acquisition protocol included bilateral lower-limb markers, force plates, inertial measurement units, electromyography sensors, and goniometers [14]. In this study, selected walking trials were used to increase the diversity of kinematic and dynamic patterns available during the development of the dynamic prediction model. Camargo/AddBiomechanics data were screened at the trial level before inclusion. Only normal level-ground walking trials were retained, while ramp and stair trials were excluded. Eligible trials were required to contain at least 120 frames, a valid GRF proportion of at least 15%, an estimated body weight greater than 500 N, and the canonical marker and dynamic-signal information required to construct the common 36-feature kinematic representation. Because GroundLink and Camargo/AddBiomechanics differ in marker configuration, data organization, sampling characteristics, and available variables, a harmonization step was required to construct shared kinematic representations. This allowed Camargo/AddBiomechanics data to contribute to the learning of GRF- and contact-related behavior while preserving GroundLink as the main reference for synchronized GRF and CoP evaluation.

GaitRec dataset. GaitRec was used as the data source for training Model 2, the gait health-status classifier [16]. Unlike GroundLink and Camargo/AddBiomechanics, GaitRec is not a kinematics-to-dynamics dataset; rather, it is a large-scale clinical gait dataset composed of bilateral GRF and CoP recordings from healthy controls and patients with musculoskeletal impairments. The dataset includes 2295 participants, comprising 211 healthy controls and 2084 patients with gait disorders involving the hip, knee, ankle, or calcaneus [16]. It contains 75,732 bilateral walking trials recorded during clinical gait assessment [16]. The available dynamic signals include vertical, anteroposterior, and mediolateral GRF components, together with anteroposterior and mediolateral CoP coordinates for the left and right sides. The processed data are normalized over stance to 101 points, which makes them suitable for machine-learning classification [16]. For this reason, the input structure of Model 2 was defined from GaitRec as a 101 × 10 representation composed of five dynamic channels per side. Consequently, the bridge block of the proposed framework was designed to transform the continuous outputs of Model 1 into this GaitRec-based 101 × 10 structure before classification.

Move AI/Blender data. Markerless kinematic data obtained with Move AI and processed in Blender were used for the final proof-of-concept deployment. These data were acquired independently from the public datasets and were not used to train the main models. In this stage, five healthy male participants were processed using the Move AI/Blender workflow. Move AI was used to generate markerless motion-capture reconstructions, and Blender was used to extract the kinematic information required by the pipeline [5,8,20]. The exported data were converted into three compatible files per trial: global equivalent points, local equivalent points, and equivalent joint angles. These files were then used to build the markerless-like input representation required by the robust version of Model 1. The Move AI/Blender trials did not include synchronized force-plate measurements. Therefore, this data source was not intended for quantitative validation of the predicted GRF and CoP against measured kinetic signals. Instead, it was used to evaluate whether the complete pipeline could operate with real markerless kinematic inputs. In this sense, the Move AI/Blender stage was treated as a functional proof of concept, while quantitative evaluation of dynamic prediction remained based on datasets containing measured GRF and CoP.

No participants were shared across the datasets used in the different stages of the framework. GroundLink and selected Camargo/AddBiomechanics participants were used only for the development and evaluation of Model 1 and the robustness analysis. GaitRec was used exclusively for the development and evaluation of Model 2. The Move AI/Blender participants were acquired independently and were not used for training either model. Within GroundLink, leave-one-subject-out evaluation was used for testing Model 1, with the remaining GroundLink participants and the selected Camargo/AddBiomechanics data used for training; an internal validation split was applied within each training fold. For Model 2, the predefined GaitRec training and held-out test partitions were respected, preserving the intended separation between model development and final evaluation.

### 2.3. Kinematic Preprocessing and Feature Harmonization

Because the datasets used in this study were obtained from different acquisition systems and followed different marker or skeletal conventions, a progressive harmonization strategy was required before model training and deployment [7,14,16,21]. The purpose of this stage was to transform heterogeneous kinematic sources into representations that could be used consistently across the different components of the proposed framework. This process evolved according to the requirements of each stage: first for the baseline kinematics-to-dynamics model, then for the robustness analysis under markerless-like conditions, and finally for the Move AI/Blender proof-of-concept deployment.

In the initial development of Model 1, the main harmonization step consisted of mapping the available kinematic information into a common set of canonical lower-limb markers. Since GroundLink and Camargo/AddBiomechanics used different marker configurations and data structures, equivalent marker trajectories were constructed to represent the anatomical regions required for gait analysis, including pelvis, hip, knee, ankle, heel, and toe-related references. This allowed both datasets to contribute to the development of the dynamic prediction model while preserving a consistent input dimensionality.

During the robustness analysis, the kinematic representation was further adapted to approximate the type of information that could be obtained from markerless motion capture [5,11,20]. For this purpose, additional equivalent points and equivalent joint angles were computed from the available anatomical references. The equivalent points represented the main lower-limb segments, whereas the equivalent angles described bilateral hip, knee, and ankle motion. This extension allowed the model to move from a purely marker-based representation toward a more general kinematic description compatible with markerless skeletal outputs. Both global and local kinematic representations were generated during this stage. The global representation preserved the spatial position of the equivalent points in the acquisition coordinate system. The local representation was obtained by expressing the lower-limb points relative to a single pelvis reference. When two pelvis-related references were available, such as right and left pelvis positions, a unique pelvis point was computed as their midpoint. This pelvis-centered representation described the lower-limb configuration relative to the body, rather than only in the absolute laboratory coordinate system. However, global motion information was not discarded; it was reintroduced later through the pelvis’ global position and velocity.

This progression led to the F3G representation used in the final robustness configuration and in the Move AI/Blender deployment. F3G combined equivalent joint angles, local equivalent points, global pelvis position, and global pelvis velocity. Therefore, the representation preserved local lower-limb configuration while also incorporating global progression cues related to body displacement during walking. This representation was used to adapt the dynamic prediction model to inputs closer to those obtainable from markerless motion capture.

Temporal handling was also adapted according to the stage of the pipeline. For datasets with synchronized kinematic and kinetic measurements, temporal consistency was maintained within each dataset during sample construction. Additional resampling was applied when needed to make the Move AI/Blender data compatible with the robust model input, particularly because the markerless data were originally obtained at 60 Hz and later converted to the temporal structure required by the deployed pipeline.

For the Move AI/Blender proof-of-concept, the same harmonization logic used in the robustness stage was applied to real markerless data. Blender outputs were converted into three compatible files for each trial: global equivalent points, local equivalent points, and equivalent joint angles. These files were then used to construct the F3G input required by the robust version of Model 1. In this way, the harmonization strategy served as the methodological link between laboratory datasets, markerless-like robustness analysis, and the final deployment using accessible markerless motion-capture data.

### 2.4. Model 1: Kinematics-to-Dynamics Prediction

Model 1 was developed to estimate gait dynamics from kinematic information. The model receives a temporal window of lower-limb marker trajectories and predicts the subsequent dynamic response of both feet. In the final baseline configuration, the kinematic input consisted of 12 canonical markers represented by their three-dimensional coordinates, resulting in 36 input features per frame. These markers were selected to preserve the main lower-limb and pelvis information required for gait analysis while maintaining compatibility between GroundLink and Camargo/AddBiomechanics data.

The regression target was defined as a set of ten bilateral dynamic signals. For the left foot, the predicted outputs were vertical, anteroposterior, and mediolateral ground reaction force components, followed by anteroposterior and mediolateral center of pressure coordinates. The same five variables were predicted for the right foot. Therefore, the final output order was: F_V_L, F_AP_L, F_ML_L, CoP_AP_L, CoP_ML_L, F_V_R, F_AP_R, F_ML_R, CoP_AP_R, and CoP_ML_R. This fixed order was preserved throughout the complete framework to ensure compatibility with the bridge block and the classification model.

Before training, the dynamic targets were biomechanically normalized to reduce inter-subject variability and improve numerical stability [6,16]. Ground reaction forces were normalized using an estimated body weight (BW_est_), expressed as force in newtons and calculated from the vertical force during support [6,16]. For GroundLink, the vertical forces of both feet were summed, and support frames were defined as those in which the total vertical force exceeded 10% of its maximum value. BW_est_ was calculated as the median total vertical force over these support frames. For Camargo/AddBiomechanics, BW_est_ was calculated as the median sum of the absolute left and right vertical forces after invalid-force frames had been removed. Trials with BW_est_ ≤ 500 N were excluded. These dataset-specific estimation procedures were required because GroundLink and Camargo/AddBiomechanics differ in data organization and contact labeling. The six GRF channels were subsequently divided by their corresponding BW_est_. On the other hand, CoP trajectories were normalized using an estimated foot length obtained from heel-to-toe marker distance [22,23]. This normalization allowed dynamic signals from different subjects and datasets to be represented in comparable units. During the final hybrid configuration, Camargo/AddBiomechanics data were allowed to contribute to the learning of GRF and contact behavior; however, the CoP loss was computed only using GroundLink samples because of representation and coordinate inconsistencies detected between datasets.

Training samples were generated using a sliding-window strategy [24]. Each input sample consisted of 30 consecutive kinematic frames. From this input window, the model predicted a future dynamic window of 30 frames. A stride of one frame was used to increase the number of training samples obtained from each trial. In addition to the predicted dynamic windows, the starting indices of each sample were preserved so that continuous trial-level predictions could later be reconstructed by averaging overlapping predictions assigned to the same temporal frame.

Two neural network architectures were evaluated for Model 1. The first architecture was based on a long short-term memory network (LSTM), using a recurrent layer with 128 hidden units followed by fully connected output heads [25]. The second architecture was a one-dimensional convolutional neural network (CNN1D), composed of two temporal convolutional layers with 64 and 128 filters, followed by adaptive average pooling and fully connected output heads. Both architectures were implemented with the same multitask output structure to allow a fair comparison between recurrent and convolutional temporal modeling. These architectures were selected because gait kinematics and dynamics constitute multivariate temporal signals. LSTM networks can retain information across successive time steps and are therefore suitable for modeling dependencies throughout the gait sequence, whereas CNN1D networks use temporal convolution kernels to extract local patterns shared across multiple channels. Recent gait studies have successfully applied these architectures to GRF and CoP estimation and to the prediction of continuous gait trajectories [26,27].

The final formulation of Model 1 used a multitask strategy. In addition to the ten regression outputs corresponding to GRF and CoP, the model predicted two binary contact signals, one for each foot. These auxiliary contact outputs were included to encourage the model to learn the temporal distinction between stance and non-stance regions. The total training loss combined the regression loss for the dynamic signals, the binary contact classification loss, and an auxiliary input-reconstruction loss. The contact loss was weighted using λ_cls = 0.1, and the reconstruction loss was weighted using λ_rec = 0.05. This formulation was adopted because it provided a more stable representation of the dynamic response while preserving the primary objective of GRF and CoP estimation.

The final training configuration used the hybrid GroundLink–Camargo setup, with six GroundLink subjects and eight selected Camargo/AddBiomechanics subjects under normal walking conditions. The final number of auxiliary subjects was selected empirically by comparing configurations containing 1, 4, 8, and 12 Camargo subjects while maintaining GroundLink as the evaluation domain. The eight-subject configuration was retained because it provided the best overall performance for the LSTM model, whereas the twelve-subject configuration produced poorer generalization toward GroundLink. Evaluation was performed using a leave-one-subject-out strategy over GroundLink, so that each GroundLink subject was used as the test subject once, while the remaining GroundLink subjects and the selected Camargo/AddBiomechanics data were used for training. Within each training fold, an internal validation split was used for early stopping, with a patience of eight epochs and a minimum improvement threshold of 1 × 10^−4^. Input features and regression targets were standardized within each fold, and the corresponding model checkpoints and preprocessing scalers were saved for subsequent stages of the framework.

After comparing the LSTM and CNN1D configurations, the LSTM-based multitask model was selected as the final Model 1 baseline. This model was then used as the dynamic prediction reference for the later stages of the pipeline, including temporal reconstruction, bridge-block processing, robustness analysis, and markerless proof-of-concept deployment.

### 2.5. Model 2: Dynamics-to-Classification Network

Model 2 was developed as the classification stage of the proposed framework. Its purpose was to classify gait health status from dynamic signals, using the output structure required by the GaitRec-based classification dataset. Unlike Model 1, which estimates dynamic variables from kinematics, Model 2 assumes that the dynamic representation has already been segmented, processed, and temporally normalized. Therefore, this model constitutes the second stage of the complete kinematics–dynamics–classification workflow.

The input to Model 2 was defined as a fixed 101 × 10 matrix. Each sample contained 101 normalized stance points and ten dynamic channels: F_V_L, F_AP_L, F_ML_L, CoP_AP_L, CoP_ML_L, F_V_R, F_AP_R, F_ML_R, CoP_AP_R, and CoP_ML_R. This order corresponds to three GRF (Ground Reaction Forces) components and two CoP (Center of Pressure) components for each foot. The use of this fixed channel order was essential because the bridge block later had to transform the continuous outputs of Model 1 into exactly the same structure before classification.

The classification target was binary. Healthy control samples were encoded as HC = 0, whereas all non-healthy-control samples were encoded as non-HC = 1. The GaitRec metadata columns were used to select the predefined training and test partitions. For training, the balanced training split was used in order to reduce the effect of class imbalance [28,29]. The test set was preserved for final evaluation, and an additional balanced test subset was used to compare the classification behavior across classes more fairly.

The base architecture of Model 2 consisted of a multichannel CNN applied to the 101 × 10 dynamic signal [17,24]. This architecture was selected because one-dimensional convolutions can identify local temporal waveform features in multichannel gait signals while preserving their sequential organization. Compact CNN1D models have recently been applied to distinguish healthy and disordered gait directly from GRF sequences [30]. The signal branch included three one-dimensional convolutional blocks with 64, 128, and 128 filters, respectively, using kernel sizes of 7, 5, and 3. Each convolutional block used rectified linear activation, L2 regularization, batch normalization, and temporal downsampling when applicable. After the convolutional feature extraction stage, global average pooling and dropout were applied to obtain a compact representation of the dynamic gait pattern.

When metadata were included, a second branch was added to the network. Numerical and categorical metadata were preprocessed separately and then concatenated into a single metadata vector. This vector was passed through a dense layer and then merged with the signal representation before the final classification layers. The final output was a single sigmoid neuron representing the probability of the non-HC class. The model was trained using binary cross-entropy loss and the Adam optimizer, with early stopping based on validation loss [24].

Three classification variants were evaluated. Variant A used only the 101 × 10 dynamic signals and served as the signal-only baseline. Variant B used the same dynamic signals together with minimal metadata: SEX, HEIGHT, and BODY_WEIGHT. In the final implementation, HEIGHT and BODY_WEIGHT were treated as numerical variables, while SEX was encoded as a categorical variable. Variant C used dynamic signals together with richer metadata available in the original dataset, including additional numerical and categorical descriptors such as age, body mass, shoe size, walking speed, affected side, footwear condition, orthopedic insole use, readmission, session type, and acquisition date-derived information. A complete description of the numerical and categorical variables included in the rich-metadata configuration, together with their anthropometric, clinical, or acquisition context, is provided in Table S1.

The comparison among these three variants was not intended only to maximize classification performance. It was also used to determine which classifier could be realistically integrated into the complete pipeline. The rich-metadata variant was useful as an upper-performance reference because it allowed the model to use a broader clinical and acquisition context. However, those variables cannot be guaranteed in a practical deployment scenario based on Move AI, Blender, and predicted dynamics from Model 1. For this reason, the minimal-metadata variant was selected as the operational classifier for the complete framework. This configuration preserved the use of subject-level information while relying only on variables that are realistically available in the proposed workflow.

To ensure reproducibility and allow later integration with the bridge block and the Move AI/Blender proof-of-concept deployment, the final minimal-metadata classifier was retrained in a dedicated script that saved all necessary artifacts. These included the complete trained model, model weights, the signal imputer and scaler, the numerical metadata imputer and scaler, the categorical metadata imputer, the SEX encoder, the exact channel order, the model configuration file, training history, and test metrics. These saved artifacts were later reused to process bridge-block outputs and guarantee that predicted dynamics were transformed using the same preprocessing operations applied during Model 2 training.

Finally, to examine the relative contribution of the dynamic signals and rich metadata in Variant C, a post hoc subject-level permutation importance analysis was performed using a retrained instance of the same architecture and preprocessing procedure. The analysis was conducted on the balanced test subset. The complete 101 × 10 dynamic representation, the complete metadata branch, and each original metadata variable were permuted separately across subjects. Subject-level donor mappings were used so that all samples from the same recipient subject received data from the same donor subject. Dynamic samples were exchanged as intact 101 × 10 matrices, without altering their temporal or channel structure, while all one-hot encoded columns belonging to the same categorical variable were permuted jointly. Each permutation was repeated 20 times. Importance was quantified as the decrease in balanced accuracy relative to the unpermuted performance of the retrained Variant C instance.

### 2.6. Bridge Block for Model Coupling and End-to-End Pipeline Evaluation

To evaluate the complete integration between Model 1 and Model 2, a bridge block was implemented within a single end-to-end pipeline. This block was necessary because the two models operate at different temporal and structural levels. Model 1 produces continuous trial-level dynamic predictions from kinematic windows, whereas Model 2 requires a fixed 101 × 10 stance-normalized dynamic representation. Therefore, the bridge block was designed as a deterministic processing stage that transforms the reconstructed outputs of Model 1 into the same type of input used during Model 2 training.

The bridge block was evaluated using two parallel dynamic branches. The first branch used the measured GroundLink dynamics, hereafter referred to as the real-dynamics branch. The second branch used the dynamics estimated by Model 1, hereafter referred to as the predicted-dynamics branch. Both branches were processed using the same support segmentation, CoP processing, temporal normalization, channel ordering, metadata preprocessing, and Model 2 classifier. This design allowed the effect of replacing measured dynamics with predicted dynamics to be evaluated under identical downstream conditions.

The first step consisted of reconstructing continuous trial-level signals from the overlapping output windows generated by Model 1. Since Model 1 was trained using sliding windows, each temporal frame could receive more than one predicted value from different overlapping windows. To obtain a single continuous prediction per trial, all predicted values assigned to the same frame were averaged. The same aggregation logic was applied to the corresponding reference dynamics, allowing real and predicted reconstructed signals to be compared under equivalent temporal conditions. This reconstruction step generated continuous signals for the ten dynamic channels and for the auxiliary left and right contact outputs.

After temporal reconstruction, the bridge block extracted support-phase segments for each foot. The vertical force channel was used to identify stance intervals. A force threshold equivalent to 20 N was used to define the presence of support, and left and right supports were processed separately [6,31,32,33]. This unilateral segmentation was important because the final Model 2 representation was based on bilateral dynamic information normalized over stance. Each extracted support segment was checked for minimum length and trimmed at the edges to reduce transition artifacts associated with initial contact and toe-off.

Specific processing was applied to the CoP channels before the final 101-point representation was generated. Because CoP is only biomechanically meaningful when the foot is in contact with the ground, a second threshold equivalent to 80 N was used to define the region where CoP was considered reliable within each support phase [6,22,23]. Outside this valid region, CoP values were not forced to zero. Instead, missing or unreliable portions were filled using interpolation, with constant extrapolation at the segment edges when necessary. This avoided introducing artificial discontinuities in the CoP trajectories. Additional CoP corrections were applied to make the reconstructed GroundLink-derived signals compatible with the distribution expected by Model 2. The anteroposterior CoP component was sign-adjusted to match the orientation convention used by the classifier input. The anteroposterior CoP was then zero-centered, whereas the mediolateral CoP was mean-centered. After the standard Model 2 preprocessing was applied, a final CoP scaling factor of α = 0.05 was applied in the scaled feature space to the CoP channels only. This calibration was used for both real and predicted branches and was part of the final frozen bridge-block configuration.

Each processed support segment was temporally normalized to 101 points using interpolation [6,16]. The final output of the bridge block was a 101 × 10 matrix with the same channel order used during Model 2 training: F_V_L, F_AP_L, F_ML_L, CoP_AP_L, CoP_ML_L, F_V_R, F_AP_R, F_ML_R, CoP_AP_R, and CoP_ML_R. This fixed structure ensured that the classifier received inputs with the same temporal length, channel order, and preprocessing convention regardless of whether the dynamics came from measured data or from Model 1 predictions.

The bridge block also incorporated the minimal metadata required by the operational version of Model 2. For each reconstructed sample, sex, height, and body weight were assigned using the subject information associated with the corresponding GroundLink trial. These metadata were processed using the same imputers, scalers, and categorical encoder saved during Model 2 training. Similarly, the dynamic signals were processed using the saved signal imputer and signal scaler from the final minimal-metadata classifier. This ensured that all bridge-block outputs were transformed consistently with the preprocessing pipeline used to train Model 2.

The final bridge-block implementation produced paired inputs for Model 2: one based on real dynamics and one based on predicted dynamics. These paired samples were stored as raw and scaled arrays, together with subject metadata, sample identifiers, labels, and configuration parameters. The third stage of the pipeline then loaded these prepared arrays and applied the same trained Model 2 to both branches. In this way, the bridge block made it possible to quantify whether the final classification output was preserved when measured dynamics were replaced by dynamics estimated from kinematics.

Therefore, the bridge block served three methodological functions. First, it converted continuous trial-level dynamic reconstructions into stance-normalized samples compatible with Model 2. Second, it standardized CoP and metadata processing between the real and predicted branches. Third, it enabled a direct end-to-end evaluation of the complete workflow, from kinematic input and dynamic prediction to final gait classification using both real and predicted dynamics.

### 2.7. Robustness Analysis Under Move AI-like Input Conditions

A robustness analysis was conducted to evaluate whether the dynamic prediction model could operate under kinematic input conditions closer to those expected from markerless motion capture. This stage was intentionally separated from the final Move AI/Blender deployment. Instead of directly applying the model to real markerless data, GroundLink and Camargo/AddBiomechanics were first transformed into a markerless-like input domain while preserving access to measured dynamic targets. This intermediate design made it possible to quantify the performance degradation caused by changing the kinematic representation before introducing the additional uncertainties associated with real markerless acquisition [5,8,11,20].

The robustness stage was built from equivalent kinematic representations. For both GroundLink and Camargo/AddBiomechanics, equivalent lower-limb points and equivalent joint angles were generated in order to approximate the type of information that could later be obtained from Move AI/Blender. The equivalent points represented pelvis, hip, knee, ankle, and foot-related landmarks, while the equivalent angles described bilateral hip, knee, and ankle motion. This transformation allowed both datasets to be expressed in a compact and shared kinematic format, independent of their original marker configurations.

Before defining the final robust representation, three feature sets were evaluated. F1 was based only on six equivalent joint angles: left hip, knee, and ankle angles, and right hip, knee, and ankle angles. F2 was based on pelvis-relative equivalent point positions, excluding the pelvis itself, resulting in 24 local position features. F3 combined both sources of information, concatenating the six joint angles and the 24 local point coordinates into a 30-feature hybrid representation. This comparison was used to determine which type of markerless-like input provided the most suitable balance between compactness, biomechanical interpretability, and predictive capacity. Based on this analysis, F3 was selected as the main representation for the subsequent robustness configuration.

The selected F3 representation was then extended into F3G. This final input included the F3 hybrid features, the global pelvis position, and the global pelvis velocity. The purpose of this extension was to preserve the local lower-limb configuration while reintroducing information related to whole-body progression during walking. This was important because a fully pelvis-relative representation may describe limb posture but may lose part of the translational information associated with forward motion and dynamic loading. Thus, F3G was used as the final Move AI-like input representation for the robust version of Model 1.

To avoid retraining the dynamic prediction model from the beginning, an adapter-based transfer strategy was implemented. The adapter received the F3G input and transformed it into a representation compatible with the input space expected by the pretrained LSTM backbone. The pretrained LSTM corresponded to the previously developed mature hybrid Model 1 configuration with the canonical marker representation. In this way, the robustness stage reused the temporal and biomechanical relationships already learned by the final dynamic prediction model, while adapting the new markerless-like input representation to that learned space.

The training procedure used GroundLink and selected Camargo/AddBiomechanics data in a hybrid configuration. Eight Camargo subjects under normal walking conditions were used as auxiliary data, while GroundLink remained the reference domain for subject-wise evaluation. The leave-one-subject-out evaluation was still performed on GroundLink, meaning that each GroundLink subject was used as the test subject once, whereas Camargo contributed only to the training side of the corresponding fold. Because CoP coordinate conventions and scaling were less consistent across domains, Camargo was used to support GRF and contact learning, while the CoP loss was computed only for GroundLink samples.

The robust model was trained in two phases. In the first phase, only the adapter was trained, allowing the model to learn how to map F3G inputs toward the canonical representation expected by the pretrained LSTM. In the second phase, a light fine-tuning stage was applied to refine the complete prediction pathway without fully discarding the pretrained structure. The same windowing configuration used in Model 1 was preserved, with 30 input frames, 30 future output frames, and a stride of one frame. The model retained the multitask formulation, including regression of the ten dynamic channels and auxiliary prediction of left and right contact.

A strong alignment loss, denoted as loss_align, was incorporated during training as a central component of the robustness strategy. This auxiliary term constrained the adapter output to remain close to the canonical input representation used by the pretrained LSTM backbone. The rationale was to prevent the adapter from drifting toward an internal representation that optimized short-term task losses but became inconsistent with the feature space learned by the original dynamic model. Therefore, loss_align acted as a domain-alignment regularizer between the F3G input domain and the canonical marker-based domain [34,35,36]. In the final configuration, the alignment loss was weighted more strongly during the adapter-only phase and reduced during the fine-tuning phase.

The final robust Model 1 configuration combined F3G inputs, an adapter module, a pretrained LSTM backbone, eight auxiliary Camargo subjects, GroundLink-based CoP supervision, multitask contact prediction, and strong alignment loss. This configuration was selected because it preserved the logic of the original dynamic prediction model while allowing the input domain to move closer to markerless motion capture. The corresponding model weights, F3G scalers, canonical-input scalers, regression-target scalers, fold metadata, and performance summaries were saved as final artifacts for subsequent use in the Move AI/Blender proof-of-concept deployment.

The objective of this robustness analysis was not to outperform the original Model 1 trained with canonical laboratory markers. Rather, it was to quantify how much predictive performance could be preserved when the input was restricted to a markerless-like representation. For this reason, the robust model was compared against the final Model 1 baseline, and the observed performance degradation was interpreted as the cost of moving from laboratory marker inputs toward a more accessible kinematic representation. This comparison provided the methodological bridge between the controlled kinematics-to-dynamics model and the later deployment using real Move AI/Blender data.

Several intermediate strategies were evaluated before defining the final robust configuration. A direct adaptation based on F3 provided an initial markerless-like baseline, but its limited representation led to a larger degradation than the later F3G formulation. Additional modifications to the adapter training were also explored, including reducing the alignment weight during the second training phase and implementing a three-stage schedule in which the adapter, output heads, and full model were progressively released. These alternatives did not improve the robustness results and, in some cases, produced less stable performance. Likewise, configurations without Camargo data or with only four auxiliary Camargo subjects were useful as intermediate comparisons, but the best balance was obtained when eight selected Camargo subjects were incorporated while keeping CoP supervision restricted to GroundLink. A stricter internal validation strategy based on subject-wise splitting and stronger determinism was also tested as a methodological check, but it did not provide a sufficient improvement to replace the established training configuration. Therefore, these alternatives were treated as exploratory stages, while the final robust model retained the F3G representation, adapter-based transfer, pretrained LSTM backbone, hybrid GroundLink–Camargo training, and domain-alignment strategy.

### 2.8. Move AI/Blender Proof-of-Concept Deployment

The final stage of the proposed framework consisted of a proof-of-concept deployment using markerless kinematic data obtained with Move AI and processed in Blender. This stage was designed to evaluate whether the complete pipeline could operate outside the public laboratory datasets used for model development. Unlike the previous experiments, this stage used real markerless motion-capture data acquired by the authors; therefore, it represented the closest implementation to the intended accessible workflow of the study.

The markerless acquisition procedure was designed to generate kinematic data that could be processed by the final deployment stage of the proposed framework. Gait sequences were recorded using an iPhone 12 (Apple Inc., Cupertino, CA, USA) and processed with Move AI to generate a three-dimensional humanoid reconstruction [8,12,20]. The resulting animation was then imported into Blender, where the reconstructed skeleton was inspected, and the useful gait interval was identified. This step was important because the original animation may include preparation frames, postural adjustment, and frames after the target walking sequence. Therefore, only the frame range corresponding to the valid walking interval was retained for subsequent processing. Blender was used not only as a visualization environment, but also as a kinematic extraction tool [37]. A Python3.11 script was implemented in Blender 4.3 to export three-dimensional coordinates frame-by-frame from the Move AI armature. The script accessed specific bones of the reconstructed skeleton and extracted anatomical references corresponding to pelvis, hip, knee, ankle, and foot for both sides. In particular, pelvis references were obtained from the Hips bone, hip and knee references from the upper-leg bones, ankle references from the leg bones, and foot references from the foot bones. For each selected frame, the corresponding coordinates were transformed to world coordinates and exported into a CSV (Comma-Separated Values) file containing the complete kinematic sequence.

This Blender-based procedure was a key step of the deployment stage because it transformed the markerless reconstruction into numerical kinematic data that could be processed by the artificial intelligence pipeline. Instead of relying only on visual inspection of the animation, the workflow produced structured time series of anatomical points, making it possible to construct inputs equivalent to those used during the robustness analysis. In this way, Move AI generated the markerless reconstruction, while Blender and its Python scripting environment converted the animation into structured numerical inputs for the model.

Before applying Model 1, the exported Blender files were converted into a format compatible with the final robust configuration developed in the previous stage. This conversion was implemented as a preliminary preprocessing step and produced three files per trial: global equivalent points, local equivalent points, and equivalent joint angles. The global points preserved the spatial trajectory of the reconstructed anatomical references. The local points were obtained by expressing the lower-limb points relative to a single pelvis reference. When left and right pelvis references were available, a unique pelvis point was computed as the midpoint between both sides. During this conversion, units, axes, signs, and sampling rate were standardized. The final coordinate convention was organized as anteroposterior, mediolateral, and vertical axes, with positive anteroposterior direction aligned with walking progression and mediolateral orientation defined consistently between sides. The exported positions were converted to millimeters when needed, and the signals were resampled from the original Move AI/Blender acquisition rate of 60 Hz (Hertz) to 250 Hz to match the temporal structure used by the robust Model 1 configuration. Equivalent joint angles were recalculated from the final standardized points, guaranteeing that the angle files and point files were generated from the same corrected geometry.

After this conversion, the F3G (F3 + global) input representation was constructed for each participant. As in the robustness analysis, F3G combined equivalent joint angles, local equivalent points, global pelvis position, and global pelvis velocity. These inputs were processed using the saved artifacts from Experiment 4 (the robustness analysis under Move AI-like input conditions described in Section 2.7), including the trained robust Model 1 folds and their corresponding scalers. Inference was performed using the same temporal structure as in the robust model: 30 input frames, 30 future prediction frames, and overlapping windows with a stride of one frame. The predicted windows were then reconstructed into continuous trial-level signals by averaging overlapping predictions assigned to the same temporal frame. The output of this stage consisted of continuous predicted dynamic signals for the ten channels used throughout the framework: F_V_L, F_AP_L, F_ML_L, CoP_AP_L, CoP_ML_L, F_V_R, F_AP_R, F_ML_R, CoP_AP_R, and CoP_ML_R. In addition, the auxiliary left and right contact probabilities were reconstructed. These outputs were saved for each participant and used as the input to the deployment version of the bridge block.

For the proof-of-concept deployment, support-phase selection was performed manually from the Blender animation instead of relying on automatic detection from the predicted force or contact signals. This decision was made because the foot–ground contact events could be directly identified from the reconstructed markerless animation, whereas the predicted contact probabilities were not intended to serve as the only reference for segmentation in this real markerless setting. The original Blender frame intervals corresponding to right and left support were therefore recorded for each participant and converted to the equivalent indices after resampling to 250 Hz. The support intervals were identified by a single evaluator through visual inspection of foot–ground contact in the Blender animations. The exact original-frame intervals used for each participant are provided in Table S2 to facilitate reproducibility.

The manual bridge block used these support intervals to extract the relevant predicted dynamic segments from each reconstructed trial. Each segment was processed to generate the final 101 × 10 representation required by Model 2. The processing steps followed the same logic established in the full-pipeline evaluation: CoP (Center of Pressure) values were treated only within valid support regions, unreliable regions were handled by interpolation, the anteroposterior CoP sign was adjusted, CoP channels were recentered, and the final support signals were temporally normalized to 101 points. The same fixed channel order used during Model 2 training was preserved.

The resulting 101 × 10 samples were then combined with the minimal metadata required by the operational classifier: sex, height, and body weight. In this proof-of-concept stage, five healthy male participants were processed. Their metadata were passed through the same preprocessing objects saved during Model 2 training, including imputers, scalers, and the categorical encoder for sex. The final classifier was then applied using the predicted dynamics generated from Move AI/Blender kinematics.

It is important to emphasize the scope of this deployment stage. The Move AI/Blender trials did not include synchronized force-plate measurements; consequently, no direct RMSE (Root Mean Square Error) against measured GRF (Ground Reaction Forces) or CoP could be computed for this stage. Therefore, the proof of concept was not intended to validate the absolute biomechanical accuracy of the estimated dynamic curves. Its purpose was to demonstrate operational feasibility: real markerless kinematics could be converted into F3G inputs, processed by the robust Model 1, transformed by the manual bridge block into 101 × 10 dynamic samples, and finally classified by Model 2. Accordingly, this stage should be interpreted as a preliminary deployment test of the complete workflow rather than as a definitive clinical or kinetic validation. Quantitative validation of GRF and CoP estimated from Move AI data would require synchronized acquisition with force plates or another kinetic reference system. Likewise, evaluation in pathological participants remains necessary to determine whether the same markerless deployment preserves discriminative capacity beyond healthy-control cases.

### 2.9. Evaluation Protocol and Metrics

The evaluation metrics were defined according to the role of each experimental stage.

For Model 1, the primary regression metric was the root mean square error (RMSE), computed between the measured and predicted normalized dynamic signals. RMSE was calculated globally across the ten output channels and separately for GRF (Ground Reaction Forces) and CoP (Center of Pressure) channels. The mean absolute error (MAE) was also computed as a complementary metric to describe the average magnitude of the prediction error. Since GRF signals were normalized by estimated body weight and CoP signals were normalized by estimated foot length, all regression errors were interpreted in normalized units. When reconstructed trial-level signals were evaluated, the metrics were computed after temporal reconstruction of the overlapping output windows. The auxiliary contact outputs of Model 1 were evaluated as binary classification signals. Predicted contact probabilities were thresholded to obtain left and right contact states, and contact accuracy was calculated by comparing the predicted contact sequence against the reference contact labels. This metric was reported independently from the regression errors because contact prediction was included as an auxiliary task to improve the temporal consistency of the dynamic estimates, especially during stance and non-stance transitions.

For Model 2, the classification target was binary: healthy control (HC) and non-healthy control (non-HC). The final sigmoid output of the classifier represented the predicted probability of the non-HC class, and a threshold of 0.5 was used to assign the final class label. Classification performance was evaluated using accuracy, balanced accuracy, F1-score, balanced F1-score, and confusion matrices. Balanced metrics were included because the clinical classification dataset contained class imbalance, and therefore standard accuracy alone could overestimate performance if one class dominated the predictions [28,29].

To quantify uncertainty in the comparisons, 95% bootstrap confidence intervals were estimated using 10,000 repetitions. For Model 1, the six GroundLink LOSO test subjects were resampled with replacement, and the differences between LSTM and CNN1D were evaluated using a paired subject-level bootstrap. For Model 2, balanced accuracy and macro F1-score were evaluated using a class-stratified sample-level bootstrap of the balanced test results, preserving the numbers of HC and non-HC samples in each repetition. The 2.5th and 97.5th percentiles of the bootstrap distributions defined the 95% confidence intervals. Because subject identifiers were not retained with the stored predictions for all three Model 2 variants, these classification intervals represent sample-level rather than subject-level uncertainty.

For the full pipeline evaluation, the measured- and predicted-dynamics branches were compared using classification accuracy, predicted non-HC probabilities, and agreement between their final labels. This analysis assessed the functional coupling of Model 1, the bridge block, and Model 2.

For the robustness analysis, the F3G-based version of Model 1 was compared with the final canonical-marker baseline using a paired subject-level analysis across the same six GroundLink LOSO test subjects. Direct metrics were paired by test subject, whereas reconstructed metrics were paired using the same reconstructed trial for each subject. A total of 10,000 paired bootstrap resamples were used to estimate 95% confidence intervals from the 2.5th and 97.5th percentiles. RMSE changes were expressed as the percentage difference relative to the baseline, whereas changes in contact accuracy were expressed in percentage points.

For the Move AI/Blender proof of concept, no measured GRF or CoP reference was available; therefore, the evaluation was limited to the predicted non-HC probabilities and final labels of the five healthy participants, focusing on operational feasibility and final classification behavior. Since no kinetic error metrics were computed, the resulting accuracy was interpreted only as a preliminary deployment outcome.

The experiments were executed in Google Colab (Google LLC, Mountain View, CA, USA; https://colab.research.google.com/, accessed on 14 August 2026). Model 1 and its robust F3G adaptation were implemented in PyTorch (PyTorch Foundation, The Linux Foundation, Wilmington, DE, USA; https://pytorch.org/, accessed on 14 August 2026), whereas Model 2 was implemented in TensorFlow/Keras (Google LLC, Mountain View, CA, USA; https://www.tensorflow.org/ and https://keras.io/, accessed on 14 August 2026). The Model 1 scripts were configured to use GPU acceleration when available and otherwise default to CPU execution. Because Colab hardware varies between sessions and computational profiling was not enabled during the original runs, exact computational benchmarks were not available for retrospective reporting. The trained models, weights, preprocessing objects, configurations, and evaluation outputs were saved to support subsequent inference and methodological reproducibility.

## 3. Results

### 3.1. Model 1 Validation

The final validation of Model 1 showed that the LSTM-based configuration achieved better dynamic prediction performance than the CNN1D configuration. Both architectures were evaluated under the same hybrid GroundLink–Camargo setup and with the same multitask formulation, including regression of the ten dynamic channels and auxiliary prediction of left and right contact.

In the direct prediction-window evaluation, the LSTM model obtained an overall RMSE of 0.0676, with RMSE values of 0.0656 for GRF and 0.0645 for CoP. The corresponding overall MAE was 0.0282, and the total contact accuracy was 0.9661. In contrast, the CNN1D model obtained an overall RMSE of 0.2191, GRF RMSE of 0.2265, CoP RMSE of 0.2051, and total contact accuracy of 0.9271.

After reconstruction of the continuous trial-level signals by averaging overlapping predictions, the LSTM also showed the best performance. The reconstructed overall RMSE was 0.0500, with reconstructed RMSE values of 0.0451 for GRF and 0.0479 for CoP. The reconstructed contact accuracy was 0.9736. The CNN1D model showed higher reconstructed errors, with an overall reconstructed RMSE of 0.1587 (Table 1).

The paired subject-level bootstrap analysis supported the consistency of the LSTM advantage. The overall RMSE was 0.0676 (95% CI: 0.0524 to 0.0801) for LSTM and 0.2191 (95% CI: 0.1419 to 0.3117) for CNN1D, with a paired LSTM-minus-CNN1D difference of −0.1515 (95% CI: −0.2366 to −0.0846). For the reconstructed signals, the paired difference in overall RMSE was −0.1087 (95% CI: −0.1729 to −0.0678), while the paired difference in reconstructed contact accuracy was 0.0343 (95% CI: 0.0198 to 0.0478). All paired confidence intervals excluded zero, supporting a consistent advantage for LSTM across the six LOSO test subjects.

**Figure 2:**
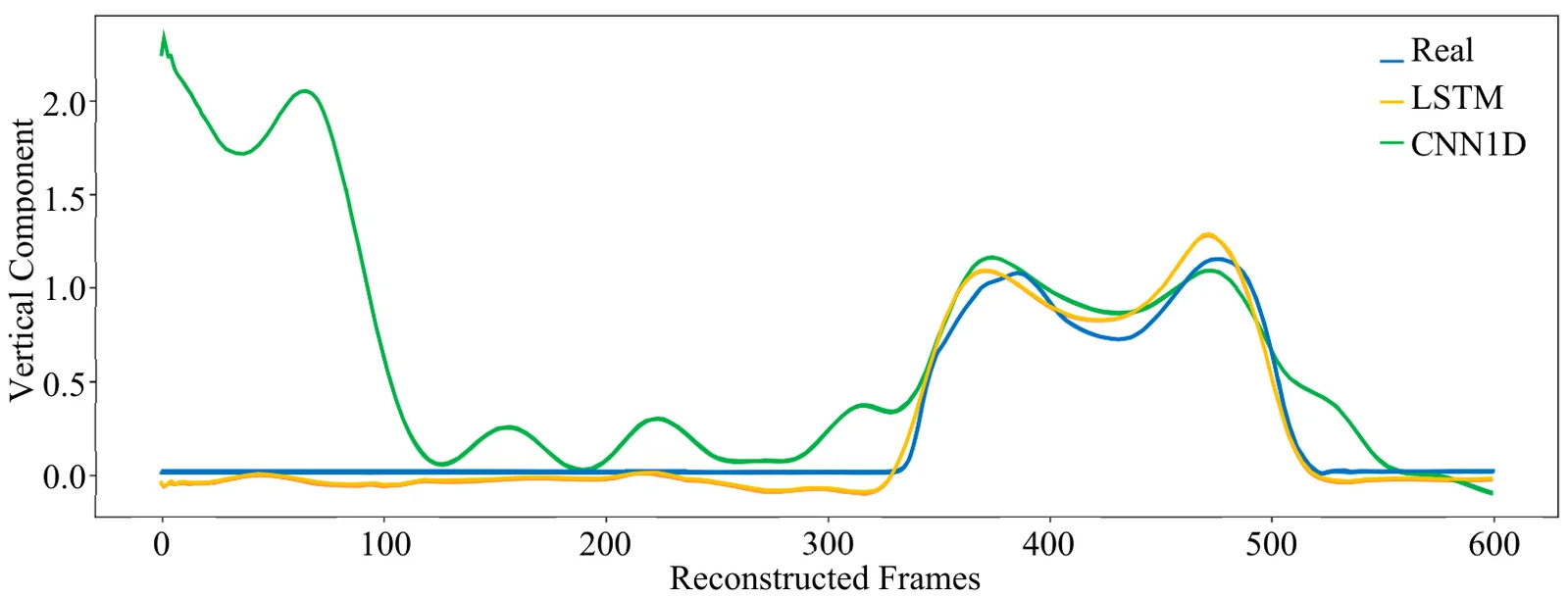
Comparison between the reconstructed measured signal and the reconstructed predicted signals of the left vertical ground reaction force component (F_V_L) for test subject s001 in Model 1 validation, using LSTM and CNN1D.

Representative reconstructed signals showed the same trend as the quantitative metrics. As shown in Figure 2 for the reconstructed F_V_L signal of test subject s001, the LSTM followed the measured vertical force profile more closely than CNN1D, particularly by reducing spurious force values in regions where the reference signal remained close to zero. Based on these results, the LSTM was retained as the final dynamic prediction architecture for the subsequent stages. Additional reconstructed examples from GroundLink test subjects representing low, intermediate, and high subject-level RMSE are provided in Figure S1, allowing visual assessment of model behavior beyond the representative s001 example.

### 3.2. Model 2 Variant Comparison

Three variants of Model 2 were evaluated using the GaitRec-based 101 × 10 dynamic representation. Variant A used only the dynamic signals, Variant B used the dynamic signals with minimal metadata, and Variant C used the dynamic signals with rich metadata. The results showed that the inclusion of metadata improved classification performance, with the strongest performance obtained when rich metadata were available.

On the balanced test subset, Variant A reached a balanced accuracy of 0.7073 (95% CI: 0.6950 to 0.7194) and a macro F1-score of 0.6947 (95% CI: 0.6810 to 0.7080). Variant B reached a balanced accuracy of 0.7548 (95% CI: 0.7420 to 0.7676) and a macro F1-score of 0.7548 (95% CI: 0.7420 to 0.7675). Variant C achieved the highest values, with a balanced accuracy of 0.9608 (95% CI: 0.9551 to 0.9662) and a macro F1-score of 0.9607 (95% CI: 0.9550 to 0.9662) (Table 2). The non-overlapping intervals support the stability of the observed performance ordering under class-stratified sample-level resampling.

Although Variant C achieved the best classification performance, Variant B was selected as the operational classifier for the complete pipeline because it used only metadata that were available in the final deployment workflow: sex, height, and body weight. Variant A was retained as a signal-only ablation.

For the operational Variant B, the confusion matrix on the balanced test subset was TN (true negatives) = 1662, FP (false positives) = 530, FN (false negatives) = 545, and TP (true positives) = 1647, considering non-HC as the positive class. These values corresponded to a sensitivity of 0.7514, a specificity of 0.7582, a false-positive rate of 0.2418, and a false-negative rate of 0.2486. These metrics characterize Model 2 independently and should not be interpreted as diagnostic performance of the complete pipeline.

**Figure 3:**
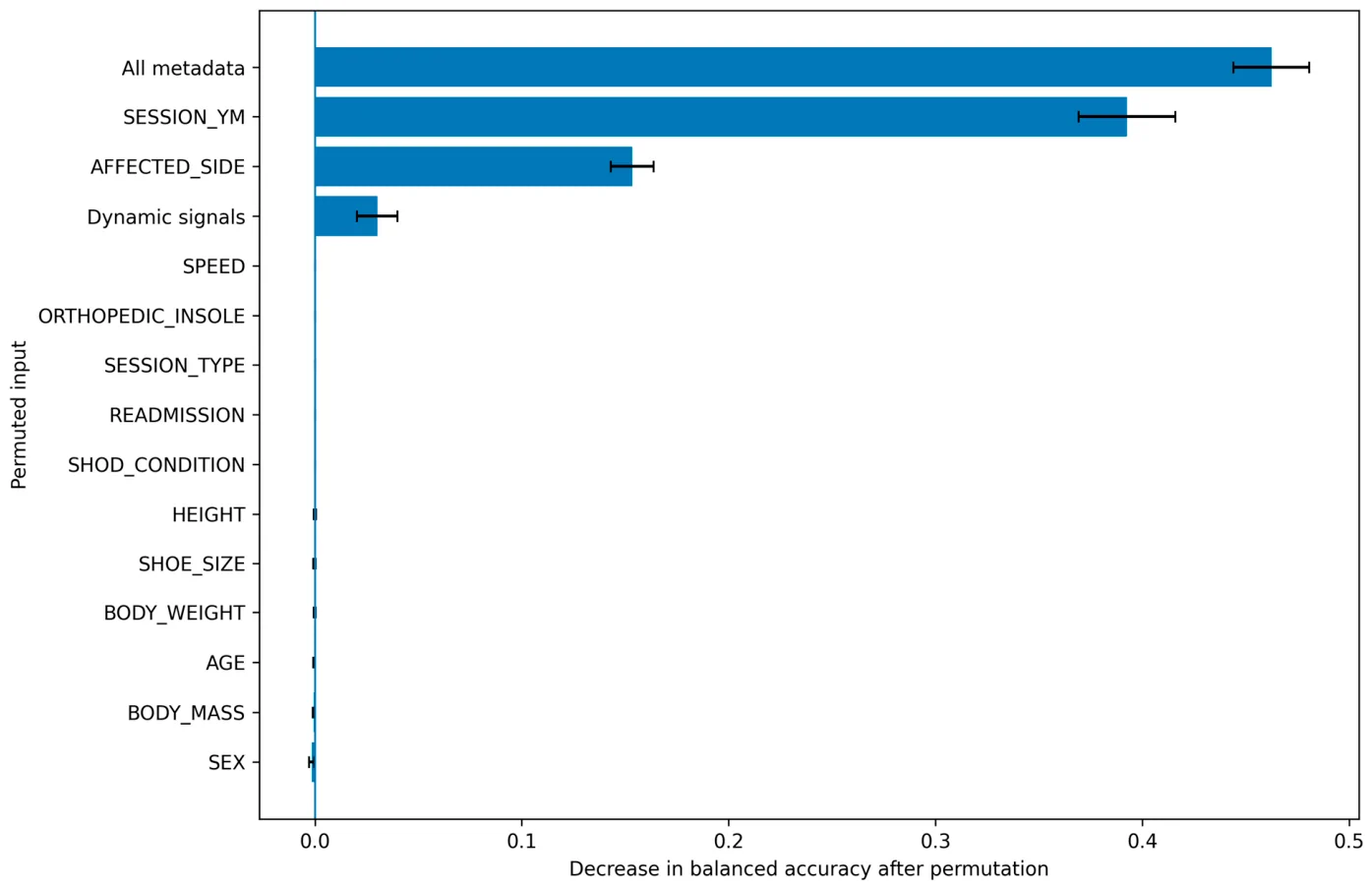
Subject-level permutation importance for the rich-metadata Model 2 configuration (Variant C). The horizontal axis represents the mean decrease in balanced accuracy after permuting each input group across subjects over 20 repetitions, while error bars indicate the corresponding standard deviation. SESSION_YM denotes acquisition year-month. Dynamic signals were permuted as intact 101 × 10 samples, and the one-hot encoded columns belonging to each categorical variable were permuted jointly. Larger decreases indicate greater model dependence on the corresponding input.

The post hoc permutation importance analysis provided additional information about the performance of the rich-metadata configuration. The retrained Variant C instance achieved a baseline balanced accuracy of 0.9859 on the balanced evaluation subset. Permuting the complete metadata branch reduced the mean balanced accuracy to 0.5234, corresponding to a mean decrease of 0.4624. The largest individual contribution was observed for the acquisition year-month variable (SESSION_YM), whose permutation produced a mean decrease of 0.3926, followed by AFFECTED_SIDE, with a decrease of 0.1533. Permuting the complete dynamic-signal branch produced a smaller but positive mean decrease of 0.0301. Conventional demographic and anthropometric variables, including age, sex, height, body weight, body mass, and shoe size, produced changes close to zero (Figure 3). Because this analysis used a retrained instance of Variant C, its baseline performance was used only as the internal reference for the permutation decreases and does not replace the primary Variant C result reported in Table 2.

### 3.3. Full Pipeline Evaluation

The complete pipeline was evaluated by comparing two parallel branches: one using measured dynamics and one using dynamics predicted by Model 1. Both branches were processed through the same bridge block and classified using the selected minimal-metadata version of Model 2. The evaluation included 17 healthy-control samples.

Both branches classified all 17 samples as healthy control. Therefore, the measured-dynamics branch and the predicted-dynamics branch both reached an accuracy of 1.0000. The agreement between both branches was also 1.0000 (Table 3).

Because the full pipeline evaluation included only healthy-control samples, sensitivity, false-negative rate, and ROC-AUC could not be calculated for this experiment. For the available HC samples, specificity was 1.0000 and the false-positive rate was 0.0000; however, without non-HC cases, these values cannot characterize discrimination between the two classes. Therefore, the reported accuracy of 1.0000 indicates correct HC classification and complete agreement between the measured- and predicted-dynamics branches, but it does not demonstrate discrimination between HC and non-HC gait.

**Figure 4:**
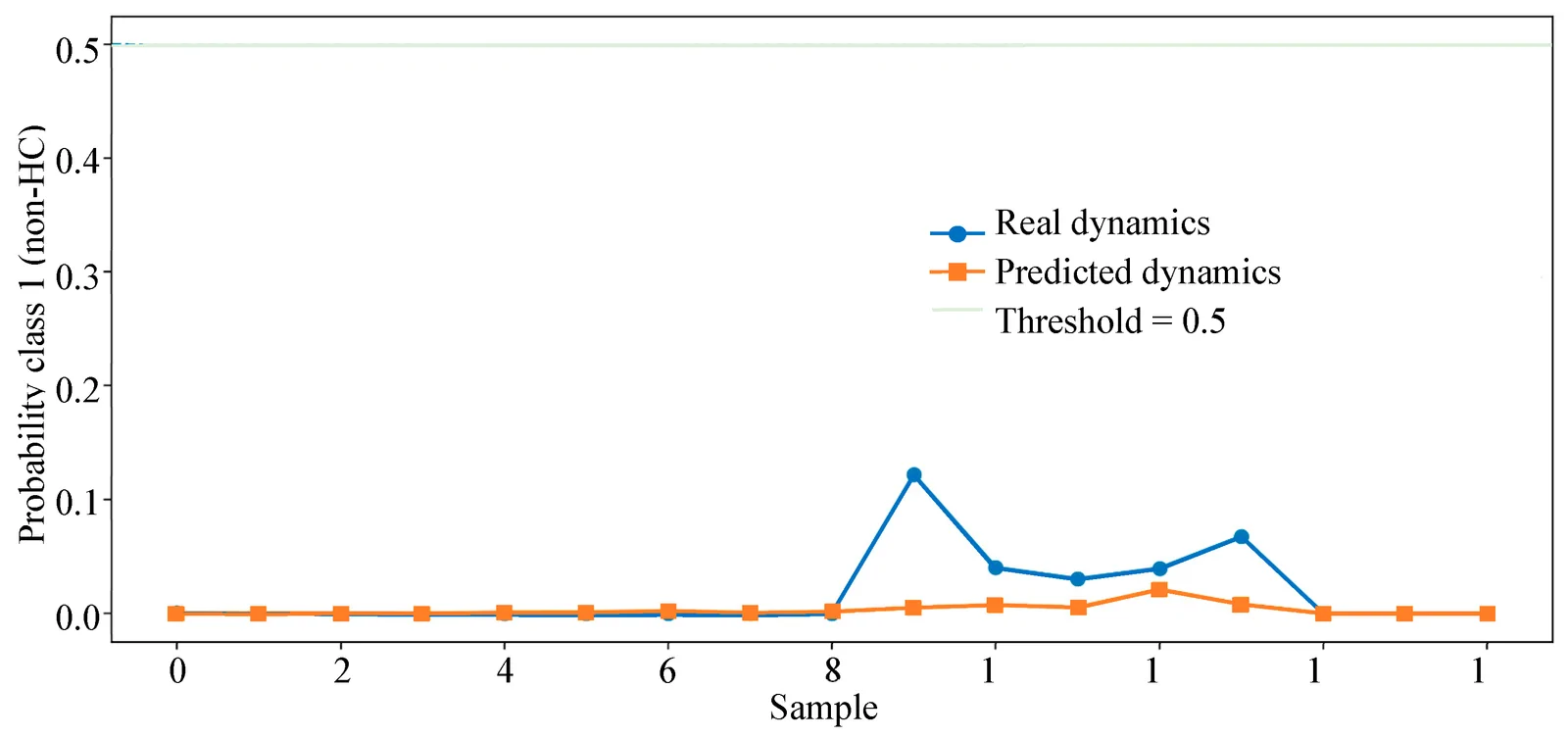
Output probabilities of Model 2 in the full pipeline evaluation using measured dynamics and predicted dynamics as input.

The predicted probabilities for the non-HC class remained below the classification threshold of 0.5 in all samples. The measured-dynamics branch produced a mean non-HC probability of 0.0177, while the predicted-dynamics branch produced a mean non-HC probability of 0.0032. These results confirm that the bridge block generated classifier-compatible inputs from both measured and predicted dynamics for the evaluated healthy-control samples. The probability-level comparison between the measured-dynamics and predicted-dynamics branches is shown in Figure 4. In both branches, the non-HC probabilities remained below the 0.5 classification threshold for all samples, indicating that replacing measured dynamics with predicted dynamics did not alter the final classification.

### 3.4. Robustness Under Move AI-like Conditions

**Figure 5:**
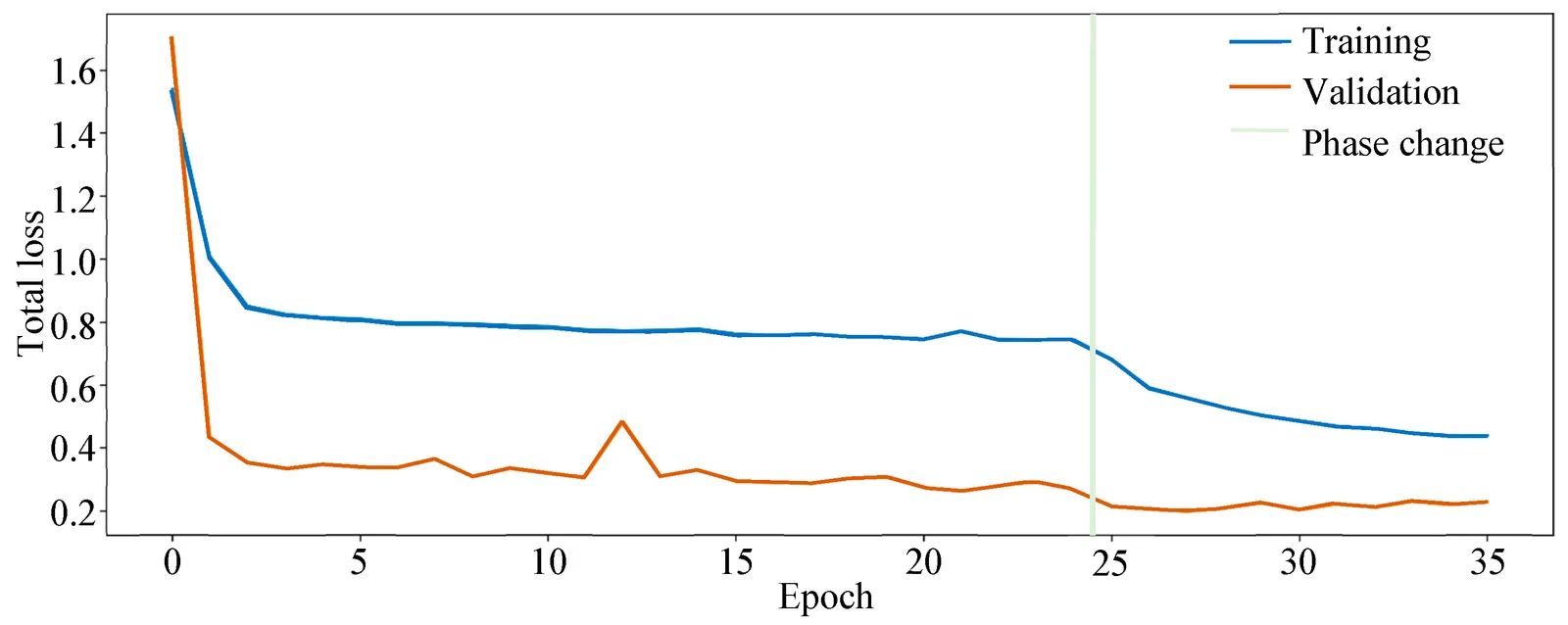
Training curves for the final robustness configuration using F3G inputs, Camargo8 auxiliary data, hybrid GroundLink–Camargo training logic, and strong loss_align, indicating the transition between training phases.

The robustness analysis evaluated the dynamic prediction model under markerless-like kinematic input conditions. The initial comparison of kinematic representations showed that the hybrid F3 representation was the most suitable base among the tested markerless-like feature sets. This representation was then extended to F3G by incorporating global pelvis position and pelvis velocity. The final robust configuration used F3G inputs, an adapter module, the pretrained LSTM backbone, selected Camargo data, and domain-alignment training. Its performance was compared against the final baseline Model 1 obtained with the canonical marker-based input representation. The training behavior of the final robust configuration is shown in Figure 5. The phase-change marker separates the adapter-only stage from the fine-tuning stage, illustrating the two-step optimization used to align the F3G representation with the pretrained LSTM backbone.

In the direct prediction-window evaluation, the robust F3G configuration reached an overall RMSE of 0.0652, GRF RMSE of 0.0712, CoP RMSE of 0.0527, overall MAE of 0.0296, and contact accuracy of 0.9659. These values were close to the final baseline Model 1, which obtained an overall RMSE of 0.0676 and contact accuracy of 0.9661.

In the reconstructed continuous-signal evaluation, the robust model reached an overall RMSE of 0.0582, GRF RMSE of 0.0636, CoP RMSE of 0.0451, MAE of 0.0267, and contact accuracy of 0.9681. The baseline Model 1 obtained reconstructed RMSE values of 0.0500 overall, 0.0451 for GRF, and 0.0479 for CoP, with reconstructed contact accuracy of 0.9736 (Table 4).

The paired subject-level analysis showed that the effect of replacing canonical marker inputs with the F3G representation was not uniform across metrics. In the direct prediction-window evaluation, the overall RMSE changed by −3.5% (95% CI: −17.7% to 9.9%), the GRF RMSE by 8.5% (95% CI: −6.6% to 25.3%), and the CoP RMSE by −18.2% (95% CI: −47.9% to 7.8%). Direct contact accuracy changed by only −0.03 percentage points (95% CI: −1.14 to 1.24). For the reconstructed evaluation, which was restricted to the same trial for each of the six subjects in both configurations, the overall RMSE changed by −9.0% (95% CI: −35.2% to 25.6%), the GRF RMSE by 15.9% (95% CI: −0.3% to 33.9%), and the CoP RMSE by −32.6% (95% CI: −63.3% to 22.1%). Reconstructed contact accuracy changed by 0.05 percentage points (95% CI: −3.30 to 3.89). Negative RMSE changes indicate lower error with F3G. All confidence intervals included zero, indicating that the available six-subject sample did not establish a uniform performance change, although the largest potential degradation was concentrated in reconstructed GRF.

**Figure 6:**
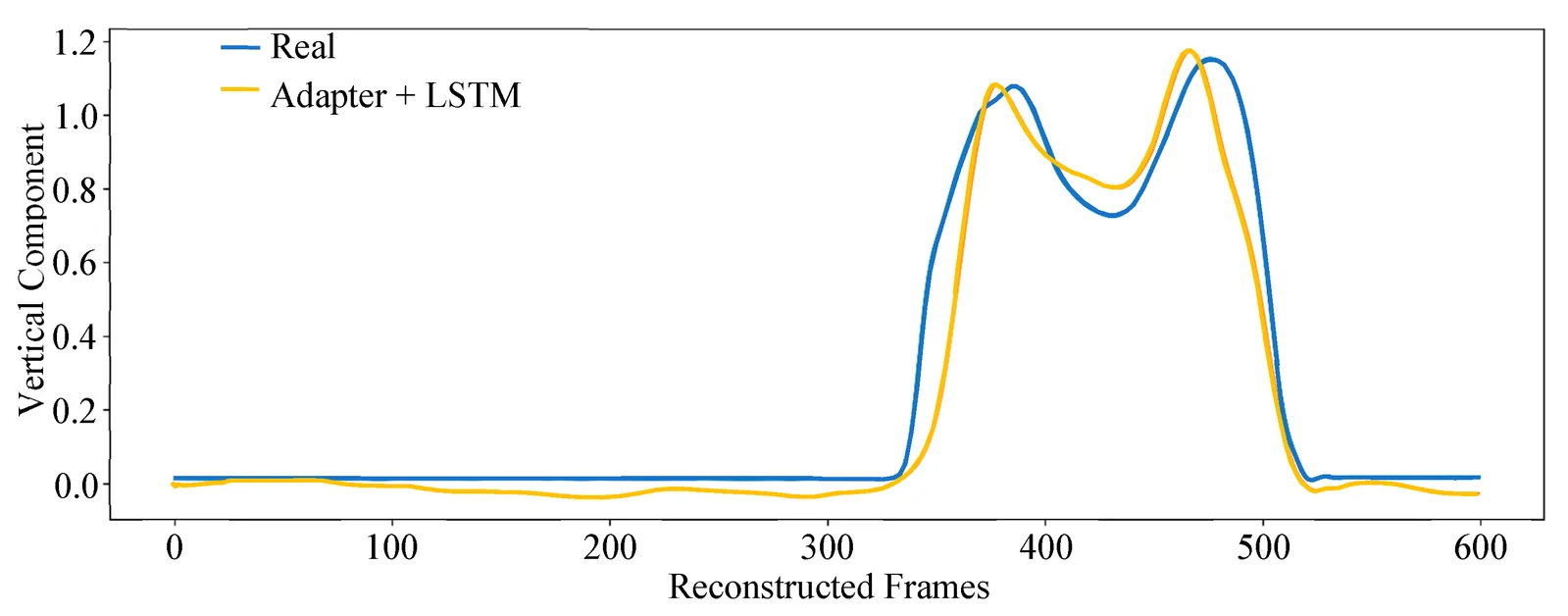
Comparison between the reconstructed measured signal and the reconstructed predicted signal of the left vertical ground reaction force component (F_V_L) for trial s001_20220513_walk_1 in the robustness analysis, using the Adapter + LSTM configuration.

This configuration was therefore used for the subsequent Move AI/Blender deployment. A representative reconstructed F_V_L signal from the robust adapter-based configuration is shown in Figure 6. The predicted curve preserved the general support-phase profile of the measured signal, although local amplitude differences and timing deviations were still observed, consistent with the reconstructed GRF error reported in Table 4.

### 3.5. Move AI/Blender Proof of Concept

The final proof-of-concept deployment was performed using markerless kinematic data obtained with Move AI and processed in Blender. Five healthy male participants were included. For each participant, the markerless kinematics were converted into F3G-compatible inputs, processed by the robust Model 1, transformed into 101 × 10 dynamic samples through the manual bridge block, and classified using the selected Model 2 variant.

All five samples were classified as healthy control. Since all participants in this stage belonged to the healthy-control group, the resulting accuracy was 1.0000. The predicted non-HC probabilities were low for all participants, with a mean value of 3.26 × 10^−6^ and a maximum value of 1.60 × 10^−5^ (Table 5).

No measured GRF or CoP signals were available for the Move AI/Blender trials. Therefore, RMSE and MAE against measured dynamics were not computed for this deployment stage.

## 4. Discussion

This study proposed and evaluated a dual-model artificial intelligence framework for gait analysis, designed to connect kinematic acquisition, dynamic prediction, and gait health-status classification within a single workflow. The main findings can be summarized in five points. First, Model 1 demonstrated that ground reaction forces (GRF) and center of pressure (CoP) can be estimated from kinematic inputs with acceptable error levels, particularly when using a long short-term memory (LSTM) architecture. Second, Model 2 confirmed that normalized dynamic signals can be used for gait classification, and that the inclusion of metadata improves classification performance. Third, the bridge block allowed the two models to be connected operationally by transforming continuous predicted dynamics into the 101 × 10 representation required by the classifier. Fourth, the robustness analysis showed that the dynamic prediction model could be adapted to markerless-like kinematic inputs using the F3G representation. Finally, the complete workflow was executed with real markerless kinematic data obtained with Move AI and processed in Blender, classifying five healthy-control participants correctly.

The superior performance of the LSTM model over the one-dimensional convolutional neural network (CNN1D) suggests that recurrent temporal modeling was better suited to the kinematics-to-dynamics mapping used in this study. Gait dynamics are strongly time-dependent: the force profile at a given instant is related not only to the current limb configuration, but also to the preceding and subsequent phases of support and limb progression [25,38,39]. The LSTM architecture may therefore have captured temporal dependencies more effectively than the CNN1D model, especially during transitions between stance and non-stance regions [25,40]. This is consistent with the reconstructed trial-level results, where the LSTM followed the measured vertical force profiles more closely and reduced spurious predictions in regions where the reference force was near zero.

The subject-level analysis provided additional insight into the most difficult cases (Table S3). Subject s001 showed the highest reconstructed overall RMSE (0.1034), the highest reconstructed CoP RMSE (0.1551), and the lowest contact accuracy in both the direct (0.9393) and reconstructed (0.8927) evaluations, indicating that its overall error was driven mainly by CoP estimation and contact-related behavior. In contrast, the highest reconstructed GRF RMSE was observed for s003 (0.0606). The representative low-, intermediate-, and high-error examples in Figure S1 suggest that the principal failure modes involved localized amplitude and timing deviations near initial contact and toe-off, together with occasional residual force predictions during non-contact. Nevertheless, the LSTM generally preserved the main stance profiles across subjects and remained more consistent than CNN1D.

The multitask formulation also appears to have contributed to the stability of Model 1. Predicting binary contact together with GRF and CoP provided the model with an additional biomechanical constraint, helping it distinguish between support and non-support intervals. This was important because dynamic variables such as GRF should approach zero during non-contact, while CoP is meaningful only during valid support [6,22,23]. In this sense, the contact output was not only an auxiliary classification target, but also a mechanism that helped organize the temporal interpretation of the predicted dynamic signals.

The classification results showed that the 101 × 10 dynamic representation contains discriminative information, as demonstrated by the signal-only configuration. More generally, contextual information can complement dynamic gait signals because variables related to anthropometry, walking conditions, clinical status, or acquisition characteristics may explain differences that are not fully captured by GRF and CoP alone [17,41]. However, the post hoc permutation analysis revealed that the high performance of Variant C was driven predominantly by its rich metadata branch. Permuting all metadata reduced balanced accuracy by 0.4624, whereas permuting the complete dynamic-signal branch produced a smaller decrease of 0.0301. The dominant individual variables were acquisition year-month and affected side. Acquisition year-month may act as a proxy for temporal cohort structure, acquisition conditions, or other dataset-specific factors associated with health-status labels. Similarly, affected side is closely related to the clinical status represented by the target class and may therefore function as a label-adjacent descriptor. In contrast, age, sex, height, body weight, body mass, and shoe size showed negligible permutation importance, suggesting that the principal concern was not conventional demographic confounding but rather clinical and acquisition-related confounding.

These findings do not imply that the dynamic signals lack discriminative value, since Variant A reached a balanced accuracy of 0.7073 using signals alone. Instead, they indicate that, when the rich metadata were available, Variant C relied primarily on contextual variables that made the classification task substantially easier. Consequently, the performance of Variant C should be interpreted as a context-enriched upper reference rather than as evidence of equivalent biomechanical discrimination from the gait signals themselves. This result further supports the selection of Variant B for the complete pipeline. Variant B uses only sex, height, and body weight, variables that showed negligible importance in the rich-metadata analysis and that can realistically be obtained in the proposed markerless workflow. Future classification studies should exclude or separately evaluate acquisition-related and label-adjacent metadata and should validate performance across temporally and clinically independent cohorts.

A noteworthy point is that the classification dataset used to train Model 2 was strongly dominated by impaired gait cases. Under this imbalance, a classifier could become biased toward the non-healthy-control class if not properly trained and evaluated [28,29]. For that reason, balanced metrics were essential for comparing the three Model 2 variants. Within this context, the fact that the complete pipeline correctly classified all available healthy-control samples in both the full pipeline evaluation and the Move AI/Blender proof of concept is encouraging. It suggests that, at least in the tested samples, the system did not simply default toward the pathological class. However, this observation should be interpreted cautiously because the end-to-end and deployment evaluations did not include pathological markerless cases.

The bridge block represents one of the most important methodological contributions of the work. The proposed framework is not only a combination of two independent models; it also includes a deterministic processing stage that allows the output of one model to become a valid input for the other. This was necessary because Model 1 produces continuous trial-level predictions, while Model 2 expects stance-normalized 101 × 10 dynamic samples. Without temporal reconstruction, support segmentation, CoP processing, channel ordering, and metadata preprocessing, the two models would not be directly compatible. The successful comparison between measured and predicted dynamic branches demonstrated that this intermediate stage can preserve the final classification output for the evaluated healthy-control samples.

The treatment of CoP was particularly important in the bridge block. Compared with GRF, CoP is more sensitive to contact definition, coordinate conventions, scaling, and the relationship between the foot and the global reference frame [22,23]. Evaluating or processing CoP outside support can introduce misleading values because CoP has no biomechanical meaning when the foot is not in contact with the ground. In other words, CoP becomes unstable when vertical force is low, and the transitions around initial contact and toe-off may therefore produce large apparent excursions that do not represent reliable plantar-loading progression. The bridge block used a 20 N threshold to identify support and an 80 N threshold to define the region where CoP was considered reliable. Values outside this region were completed by interpolation with constant edge extrapolation, while the anteroposterior sign convention was adjusted, the anteroposterior and mediolateral trajectories were recentered, and a final calibration factor of α = 0.05 was applied in the scaled feature space. These operations reduced artificial discontinuities and aligned the measured- and predicted-dynamics branches with the input distribution of Model 2, allowing CoP information to be retained in the standardized 101 × 10 representation.

The robustness analysis addressed the controlled domain shift between laboratory marker-based kinematics and markerless-like F3G inputs [5,11,34,35,36,42]. The final baseline Model 1 was trained using canonical marker trajectories, whereas the intended deployment relies on skeletal information extracted from markerless motion capture. This change in input representation can affect both geometry and temporal consistency [5,11,42]. The F3G representation was introduced to reduce this gap by combining local lower-limb configuration, joint angles, global pelvis position, and pelvis velocity. The robust configuration did not eliminate all performance degradation, especially in reconstructed GRF signals, but it preserved enough predictive behavior to justify its use as the deployment model for the Move AI/Blender proof of concept.

The paired analysis did not reveal a uniform performance loss across the six GroundLink subjects. Direct overall RMSE changed by −3.5%, while direct contact accuracy changed by only −0.03 percentage points. Similarly, reconstructed overall RMSE changed by −9.0%, and reconstructed contact accuracy remained essentially unchanged, with a difference of 0.05 percentage points. The main potential cost was concentrated in the reconstructed GRF signals, whose RMSE increased by 15.9% (95% CI: −0.3% to 33.9%). In contrast, no systematic degradation was observed for reconstructed CoP, although the wide confidence intervals reflect the uncertainty associated with the limited number of subjects. From a practical perspective, these results suggest that the F3G adaptation largely preserved the temporal contact information and overall dynamic-prediction behavior, while local GRF amplitudes and timing may be less faithful after reconstruction. This distinction is relevant because contact structure supports temporal reconstruction and support-phase processing, whereas detailed GRF waveform interpretation requires greater quantitative precision. Nevertheless, the confidence intervals included zero, and the findings should not be interpreted as demonstrating statistical equivalence between the two input representations. Moreover, this analysis quantified only the controlled transition from canonical markers to markerless-like F3G inputs derived from laboratory data. The additional domain shift introduced by real Move AI reconstruction remains unquantified because synchronized kinetic reference measurements were unavailable during the proof-of-concept deployment.

The use of Camargo/AddBiomechanics data during Model 1 development and robustness analysis also contributed to generalization, but introduced additional heterogeneity. GroundLink provided synchronized kinematics and measured GRF/CoP suitable for validation, whereas Camargo offered a broader source of locomotion data. This combination was useful because this way, the model was exposed to more diverse motion and force patterns. At the same time, the datasets differed in marker structure, trial segmentation, coordinate conventions, and CoP representation. For this reason, Camargo was most useful for improving GRF and contact-related behavior, while CoP supervision required a more conservative treatment centered on GroundLink.

The Move AI/Blender proof of concept demonstrated that the complete workflow can operate on real markerless kinematic data. All five healthy participants were classified as healthy controls, with non-healthy-control probabilities far below the classification threshold. This result supports the operational feasibility of the proposed workflow using accessible markerless kinematics. However, because the Move AI/Blender trials did not include synchronized force-plate measurements, this stage should be interpreted as a functional deployment test rather than as a quantitative validation of the estimated GRF or CoP. In addition, because only healthy participants were included, this proof of concept does not yet establish clinical discrimination between healthy and pathological gait.

Several limitations should be considered. The GroundLink subset used for subject-wise validation comprised only six participants, which limits the statistical robustness and generalizability of the dynamic-prediction results and leaves a risk of dataset-specific overfitting. Subject-wise LOSO evaluation and bootstrap confidence intervals were used to quantify performance variability across the available participants, but these procedures cannot compensate for the limited number of independent subjects. Although the inclusion of eight Camargo/AddBiomechanics participants increased the diversity of the training data, these participants were not evaluated as an independent external test cohort. Therefore, validation in a larger and fully independent kinematic–kinetic dataset remains necessary. Although Model 2 was trained and evaluated using both healthy-control and non-healthy-control samples from GaitRec, the full pipeline evaluation and the Move AI/Blender proof of concept were restricted to healthy-control samples. Therefore, the complete end-to-end system has not yet been evaluated across both healthy and pathological cases. This evaluation could not be performed with the available datasets because GaitRec provides pathological dynamic signals but not the compatible kinematic inputs required by Model 1, whereas the kinematic–dynamic datasets used for Model 1 contain only able-bodied participants. Furthermore, the five healthy male participants included in the Move AI/Blender stage were sufficient only for an operational proof of concept and do not support population-level generalization or estimation of clinical diagnostic performance. For the Move AI/Blender trials, the absence of synchronized kinetic reference data prevented direct validation of the predicted GRF and CoP. Domain shift also remains an important challenge, since laboratory datasets and markerless reconstructions differ in skeletal definitions, coordinate conventions, sampling characteristics, and measurement uncertainty [5,8,11,34,35,36,42]. In addition, CoP estimation remains particularly sensitive because it depends strongly on contact definition, coordinate alignment, scaling, and foot-reference consistency. The final workflow also involved several sequential processing decisions, including feature harmonization, temporal reconstruction, support selection, CoP processing, scaling, and metadata integration; each of these steps was necessary to make the pipeline operational, but each may also contribute to accumulated error. Finally, support-phase segmentation in the Move AI/Blender deployment was performed by a single evaluator; therefore, inter-rater reliability was not available, and the annotation time was not systematically recorded. This manual procedure may introduce observer dependence and limits scalability. Future implementations should evaluate automated support detection using predicted contact probabilities, vertical GRF, foot height, foot velocity, or a combination of these signals.

Future work should focus on three main directions. First, the Move AI/Blender deployment should be repeated with synchronized force plates or another kinetic reference system, allowing direct quantitative validation of the estimated GRF and CoP [13,18,38,39,40]. Second, the markerless proof of concept should be extended to pathological participants, so that the complete pipeline can be evaluated under clinically relevant gait alterations. Third, the robust Model 1 should be further adapted specifically to real Move AI data, rather than only to markerless-like representations derived from laboratory datasets. This could include domain-specific calibration, larger markerless datasets, and refinement of the F3G representation. Additional work should also explore richer clinical metadata when available. The present pipeline selected minimal metadata for practical deployment, but the strong performance of the rich-metadata classifier suggests that future clinical implementations could benefit from incorporating broader patient descriptors.

## 5. Conclusions

This study presented a dual-model artificial intelligence framework for gait analysis that connects kinematic acquisition, dynamic prediction, and gait health-status classification within a single workflow. The results showed that Model 1 can estimate bilateral ground reaction forces (GRF) and center of pressure (CoP) from kinematic inputs, with the Long Short-Term Memory (LSTM) architecture providing the best performance among the tested dynamic prediction models.

The classification stage confirmed that dynamic signals contain useful information for distinguishing healthy-control and non-healthy-control gait patterns. Although the rich-metadata configuration achieved the highest classification performance, the permutation importance analysis showed that much of its additional contribution was associated with acquisition and clinical context, particularly acquisition year-month and affected side. Therefore, the version using minimal metadata was selected for the complete pipeline because it depends only on sex, height, and body weight, which are more feasible to obtain in practical markerless acquisition scenarios.

A central contribution of the study was the implementation of the bridge block, which transformed continuous dynamic predictions into the standardized 101 × 10 input required by the classifier. This intermediate stage allowed the two models to operate as a connected system rather than as isolated components. The full pipeline evaluation showed that, for the available healthy-control samples, replacing measured dynamics with predicted dynamics preserved the final classification output.

The robustness analysis showed that the F3G adaptation largely preserved overall prediction behavior and contact accuracy, although reconstructed GRF represented the main potential source of performance degradation. The complete pipeline also successfully processed real Move AI/Blender data from five healthy participants. This deployment should be interpreted as a proof of operational feasibility rather than as complete clinical or kinetic validation, since synchronized force-plate measurements and pathological markerless cases were not available. Future work should validate the pipeline with pathological participants, simultaneous markerless and force-plate acquisition, and further adaptation to real markerless motion-capture data. Overall, the study establishes a reproducible foundation for connecting accessible kinematic acquisition with dynamic estimation and gait classification within an integrated artificial intelligence framework.

## Figures and Tables

**Table 1:** Final Model 1 performance for CNN1D and LSTM.

Model	RMSE Total	RMSE GRF	RMSE CoP	MAE Total	Contact Accuracy	Reconstructed RMSE Total	Reconstructed RMSE GRF	Reconstructed RMSE CoP	Reconstructed MAE Total	Reconstructed Contact Accuracy
CNN1D	0.2191	0.2265	0.2051	0.0842	0.9271	0.1587	0.1701	0.1299	0.0686	0.9393
LSTM	0.0676	0.0656	0.0645	0.0282	0.9661	0.0500	0.0451	0.0479	0.0221	0.9736

**Table 2:** Classification performance of the three Model 2 variants.

Variant	Input Configuration	Full Test Accuracy	Full Test Macro F1-Score	Balanced Test Accuracy	Balanced Test Macro F1-Score
A	Dynamic signals only	0.8722	0.6786	0.7073	0.6947
B	Dynamic signals + minimal metadata	0.7378	0.5955	0.7548	0.7548
C	Dynamic signals + rich metadata	0.9248	0.8367	0.9608	0.9607

**Table 3:** Full pipeline classification using measured and predicted dynamics.

Branch	Samples	Expected HC	Predicted HC	Predicted Non-HC	Accuracy	Mean Non-HC Probability	Maximum Non-HC Probability	Branch Agreement
Measured dynamics	17	17	17	0	1.0000	0.0177	0.1220	1.0000
Predicted dynamics	17	17	17	0	1.0000	0.0032	0.0212	1.0000

**Table 4:** Comparison between the final baseline Model 1 and the robust F3G configuration.

Model Configuration	RMSE Total	RMSE GRF	RMSE CoP	MAE Total	Contact Accuracy	Reconstructed RMSE Total	Reconstructed RMSE GRF	Reconstructed RMSE CoP	Reconstructed MAE Total	Reconstructed Contact Accuracy
Final baseline Model 1	0.0676	0.0656	0.0645	0.0282	0.9661	0.0500	0.0451	0.0479	0.0221	0.9736
Robust F3G configuration	0.0652	0.0712	0.0527	0.0296	0.9659	0.0582	0.0636	0.0451	0.0267	0.9681

**Table 5:** Move AI/Blender proof-of-concept classification results.

Samples	Expected Class	Predicted HC	Predicted Non-HC	Accuracy	Mean Non-HC Probability	Maximum Non-HC Probability
5	HC	5	0	1.0000	3.26 × 10^−6^	1.60 × 10^−5^

## Data Availability

The public datasets analyzed in this study are available from their original sources, including GroundLink, Camargo/AddBiomechanics, and GaitRec, as described in the corresponding dataset publications. The data generated during the Move AI/Blender proof-of-concept stage and the derived model artifacts are available from the corresponding author upon reasonable request, subject to privacy considerations and participant consent. The processed results supporting the findings of this study are reported within the article and its Supplementary Materials.

## Supplementary Materials

The following supporting information can be downloaded at: https://www.mdpi.com/article/10.3390/diagnostics16162588/s1, Figure S1: Additional reconstructed vertical ground reaction force examples for three GroundLink test subjects not shown in the main figure, selected to represent low, intermediate, and high reconstructed RMSE among the additional subjects: (a) s004, (b) s006, and (c) s003. Each panel compares the measured F_V_L signal with the LSTM and CNN1D reconstructions over the selected walking trial; Table S1: Variables included in the rich-metadata configuration of Model 2; Table S2: Original Blender frame intervals manually selected for the right and left support phases of the five Move AI/Blender participants. The final frame of each interval was treated as inclusive; Table S3: Subject-level direct and reconstructed performance of the final LSTM model across the six GroundLink leave-one-subject-out test folds. The supplementary files also include six Jupyter notebooks covering kinematic preprocessing, feature harmonization, Model 1 training and inference, bridge-block construction, Model 2 training and classification, robustness analysis, and proof-of-concept deployment, together with the trained models and preprocessing artifacts supporting the reported experiments, including scalers, imputers, encoders, channel-order definitions, configurations, and model weights.

## References

[1] Hulleck A.A., Menoth Mohan D., Abdallah N., El Rich M., Khalaf K. (2022). Present and future of gait assessment in clinical practice: Towards the application of novel trends and technologies. Front. Med. Technol..

[2] Hebda-Boon A., Tan X.L., Tillmann R., Shortland A.P., Firth G.B., Morrissey D. (2023). The impact of instrumented gait analysis on decision-making in the interprofessional management of cerebral palsy: A scoping review. Eur. J. Paediatr. Neurol..

[3] Alderink G., Õunpuu S. (2025). Biomechanics of human motion and its clinical applications: Instrumented gait analysis. Bioengineering.

[4] D’Haene M., Chorin F., Colson S.S., Guérin O., Zory R., Piche E. (2024). Validation of a 3D markerless motion capture tool using multiple pose and depth estimations for quantitative gait analysis. Sensors.

[5] Scataglini S., Abts E., Van Bocxlaer C., Van den Bussche M., Meletani S., Truijen S. (2024). Accuracy, validity, and reliability of markerless camera-based 3D motion capture systems versus marker-based 3D motion capture systems in gait analysis: A systematic review and meta-analysis. Sensors.

[6] Horst F., Slijepcevic D., Simak M., Schöllhorn W.I. (2021). Gutenberg Gait Database, a ground reaction force database of level overground walking in healthy individuals. Sci. Data.

[7] Han X., Senderling B., To S., Kumar D., Whiting E., Saito J. (2023). GroundLink: A dataset unifying human body movement and ground reaction dynamics. SIGGRAPH Asia 2023 Conference Papers (SA ’23).

[8] Wade L., Needham L., McGuigan P., Bilzon J. (2022). Applications and limitations of current markerless motion capture methods for clinical gait biomechanics. PeerJ.

[9] Lam W.W.T., Tang Y.M., Fong K.N.K. (2023). A systematic review of the applications of markerless motion capture technology for clinical measurement in rehabilitation. J. NeuroEng. Rehabil..

[10] Wishaupt K., Schallig W., van Dorst M.H., Buizer A.I., van der Krogt M.M. (2024). The applicability of markerless motion capture for clinical gait analysis in children with cerebral palsy. Sci. Rep..

[11] D’Souza S., Siebert T., Fohanno V. (2024). A comparison of lower body gait kinematics and kinetics between Theia3D markerless and marker-based models in healthy subjects and clinical patients. Sci. Rep..

[12] Uhlrich S.D., Falisse A., Kidziński Ł., Muccini J., Ko M., Chaudhari A.S., Hicks J.L., Delp S.L. (2023). OpenCap: Human movement dynamics from smartphone videos. PLoS Comput. Biol..

[13] Lichtwark G.A., Schuster R.W., Kelly L.A., Trost S.G., Bialkowski A. (2024). Markerless motion capture provides accurate predictions of ground reaction forces across a range of movement tasks. J. Biomech..

[14] Camargo J., Ramanathan A., Flanagan W., Young A. (2021). A comprehensive, open-source dataset of lower limb biomechanics in multiple conditions of stairs, ramps, and level-ground ambulation and transitions. J. Biomech..

[15] Werling K., Kaneda J., Tan T., Agarwal R., Skov S., Van Wouwe T., Uhlrich S., Bianco N., Ong C., Falisse A., Leonardis A., Ricci E., Roth S., Russakovsky O., Sattler T., Varol G. (2025). AddBiomechanics Dataset: Capturing the physics of human motion at scale. Computer Vision—ECCV 2024 Workshops.

[16] Horsak B., Slijepcevic D., Raberger A.M., Schwab C., Worisch M., Zeppelzauer M. (2020). GaitRec, a large-scale ground reaction force dataset of healthy and impaired gait. Sci. Data.

[17] Pandey C., Roy D.S., Poonia R.C., Altameem A., Nayak S.R., Verma A., Saudagar A.K.J. (2022). GaitRec-Net: A deep neural network for gait disorder detection using ground reaction force. PPAR Res..

[18] Feng R., Ugbolue U.C., Yang C., Liu H. (2025). Estimation of three-dimensional ground reaction force and center of pressure during walking using a machine-learning-based markerless motion capture system. Bioengineering.

[19] Sugiarto T., Lin Y.-J., Tsai H.-L., Sun C.-T., Hsu W.-C. (2025). Performance of deep-learning models incorporating knee alignment information for predicting ground reaction force during walking. BioMed. Eng. OnLine.

[20] Kidziński Ł., Yang B., Hicks J.L., Rajagopal A., Delp S.L., Schwartz M.H. (2020). Deep neural networks enable quantitative movement analysis using single-camera videos. Nat. Commun..

[21] Werling K., Bianco N.A., Raitor M., Stingel J., Hicks J.L., Collins S.H., Delp S.L., Liu C.K. (2023). AddBiomechanics: Automating model scaling, inverse kinematics, and inverse dynamics from human motion data through sequential optimization. PLoS ONE.

[22] Jeon E.-T., Cho H.-Y. (2020). A novel method for gait analysis on center of pressure excursion based on a pressure-sensitive mat. Int. J. Environ. Res. Public Health.

[23] Li B., Xiang Q., Zhang X. (2020). The center of pressure progression characterizes the dynamic function of high-arched feet during walking. J. Leather Sci. Eng..

[24] Foumani N., Miller L., Tan C.W., Webb G.I., Forestier G., Salehi M. (2024). Deep learning for time series classification and extrinsic regression: A current survey. ACM Comput. Surv..

[25] Alcantara R.S., Edwards W.B., Millet G.Y., Grabowski A.M. (2022). Predicting continuous ground reaction forces from accelerometers during uphill and downhill running: A recurrent neural network solution. PeerJ.

[26] Kim J., Kim K.-C., Tack G., Choi J.-S. (2025). Estimation of 3D ground reaction force and 2D center of pressure using deep learning and load cells across various gait conditions. Sensors.

[27] Khan A., Galarraga O., Garcia-Salicetti S., Vigneron V. (2024). Phase-based gait prediction after botulinum toxin treatment using deep learning. Sensors.

[28] Thölke P., Mantilla-Ramos Y.J., Abdelhedi H., Maschke C., Dehgan A., Harel Y., Kemtur A., Berrada L.M., Sahraoui M., Young T. (2023). Class imbalance should not throw you off balance: Choosing the right classifiers and performance metrics for brain decoding with imbalanced data. NeuroImage.

[29] Salmi M., Atif D., Oliva D., Abraham A., Ventura S. (2024). Handling imbalanced medical datasets: Review of a decade of research. Artif. Intell. Rev..

[30] Shafew A., Kim D., Kim D. (2024). Simple 1D CNN model for accurate classification of gait patterns using GRF data. J. Inf. Commun. Converg. Eng..

[31] Visscher R.M., Sansgiri S., Freslier M., Harlaar J., Brunner R., Taylor W.R., Singh N.B. (2021). Towards validation and standardization of automatic gait event identification algorithms for use in paediatric pathological populations. Gait Posture.

[32] Bonci T., Salis F., Scott K., Alcock L., Becker C., Bertuletti S., Buckley E., Caruso M., Cereatti A., Del Din S. (2022). An algorithm for accurate marker-based gait event detection in healthy and pathological populations during complex motor tasks. Front. Bioeng. Biotechnol..

[33] Wang S., Omar K.S., Miranda F., Bhatt T. (2025). Automatic gait event detection in older adults during perturbed walking. J. NeuroEng. Rehabil..

[34] Wilson G., Cook D.J. (2020). A survey of unsupervised deep domain adaptation. ACM Trans. Intell. Syst. Technol..

[35] Shi Y., Ying X., Yang J. (2022). Deep unsupervised domain adaptation with time series sensor data: A survey. Sensors.

[36] Zhao S., Yue X., Zhang S., Li B., Zhao H., Wu B., Krishna R., Gonzalez J.E., Sangiovanni-Vincentelli A.L., Seshia S.A. (2022). A review of single-source deep unsupervised visual domain adaptation. IEEE Trans. Neural Netw. Learn. Syst..

[37] Denninger M., Winkelbauer D., Sundermeyer M., Boerdijk W., Knauer M., Strobl K.H., Humt M., Triebel R. (2023). BlenderProc2: A procedural pipeline for photorealistic rendering. J. Open Source Softw..

[38] Martínez-Pascual D., Catalán J.M., Blanco-Ivorra A., Sanchís M., Arán-Ais F., García-Aracil N. (2023). Estimating vertical ground reaction forces during gait from lower limb kinematics and vertical acceleration using wearable inertial sensors. Front. Bioeng. Biotechnol..

[39] Cerfoglio S., Galli M., Tarabini M., Bertozzi F., Sforza C., Zago M. (2021). Machine learning-based estimation of ground reaction forces and knee joint kinetics from inertial sensors while performing a vertical drop jump. Sensors.

[40] Verheul J., Robinson M.A., Burton S. (2024). Jumping towards field-based ground reaction force estimation and assessment with OpenCap. J. Biomech..

[41] Brzenczek C., Klopfenstein Q., Hähnel T., Sapienza S., Klucken J., Fröhlich H., Glaab E. (2024). Integrating digital gait data with metabolomics and clinical data to predict outcomes in Parkinson’s disease. npj Digit. Med..

[42] Kanko R.M., Laende E.K., Davis E.M., Selbie W.S., Deluzio K.J. (2021). Concurrent assessment of gait kinematics using marker-based and markerless motion capture. J. Biomech..

