# Supplementary material for: Dual-Model Artificial Intelligence Framework Integrating AI-Based Markerless Motion Capture for Dynamic Gait Prediction and Health-Status Classification
Source: Diagnostics (Basel). 2026 Aug 16;16(16):2588. doi: 10.3390/diagnostics16162588 (PMC13511503; doi:10.3390/diagnostics16162588)
Supplement: Supplementary file 1 [file diagnostics-16-02588-s001.zip › diagnostics-4387648-supplementary.pdf]

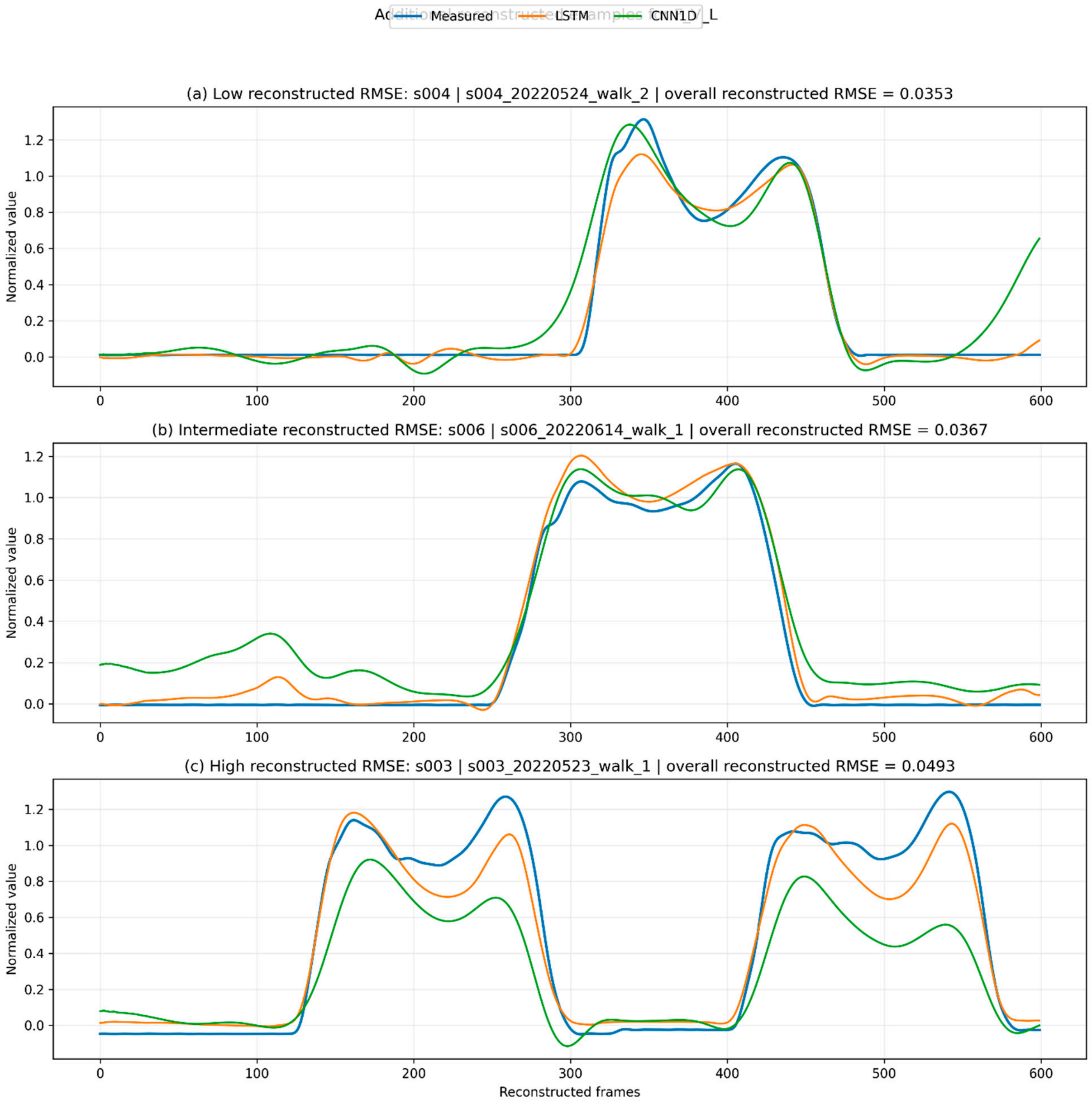

Figure S1. Additional reconstructed vertical ground reaction force examples for three GroundLink test subjects not shown in the main figure, selected to represent low, intermediate, and high reconstructed RMSE among the additional subjects: (a) s004, (b) s006, and (c) s003. Each panel compares the measured F_V_L signal with the LSTM and CNN1D reconstructions over the selected walking trial.

Table S1. Variables included in the rich-metadata configuration of Model 2.

Variable	Type in Variant C	Description and contextual relevance
AGE	Numerical	Age at the recording date, expressed in years. Age may capture differences in gait mechanics, functional capacity, and the prevalence or severity of musculoskeletal conditions.
HEIGHT	Numerical	Body height in centimeters. Height provides anthropometric context related to limb dimensions, step characteristics, and the scaling of gait patterns.
BODY_WEIGHT	Numerical	Body weight expressed in newtons. It represents the subject's gravitational load and provides context for the magnitude of measured ground reaction forces.
BODY_MASS	Numerical	Body mass in kilograms. Body mass may influence loading patterns and complements the force-based body-weight variable.
SHOE_SIZE	Numerical	Shoe size expressed in the Continental European system. It provides an indirect descriptor of foot dimensions that may be associated with contact geometry and CoP progression.
SPEED	Numerical, ordinal	Walking-speed condition: slow = 1, self-selected = 2, and fast = 3. Walking speed strongly influences GRF magnitude, support duration, propulsion, braking, and CoP trajectories.
SEX	Categorical	Biological sex encoded as female = 0 and male = 1. It provides demographic and anthropometric context that may be associated with differences in gait patterns.
AFFECTED_SIDE	Categorical	Side associated with the musculoskeletal impairment: left = 0, right = 1, and bilateral = 2. This variable provides direct clinical context regarding the location of the impairment and may be closely associated with the health-status label.
SHOD_CONDITION	Categorical	Footwear condition: barefoot or socks = 0, normal shoe = 1, and orthopedic shoe = 2. Footwear can modify plantar loading, force transmission, and CoP behavior.
ORTHOPEDIC_INSOLE	Categorical	Indicates whether an orthopedic insole was absent = 0 or present = 1. Insoles may alter load distribution, foot support, and the measured dynamic response.
READMISSION	Categorical	Number of readmissions associated with the participant's clinical record. It may provide context regarding repeated assessment, treatment history, or longitudinal clinical follow-up.
SESSION_TYPE	Categorical	Type of assessment session: initial measurement = 1, control measurement = 2, and initial measurement after readmission = 3. It describes the stage of the clinical assessment and may reflect treatment or follow-up context.

SESSION_YM	Categorical, derived	Acquisition year and month derived from SESSION_DATE. It provides temporal acquisition context but is not a biomechanical variable. It may capture cohort, protocol, equipment, or other time-related dataset characteristics.

Note: SESSION_YM was derived from the original SESSION_DATE field by retaining the year and month of acquisition. Numerical variables were imputed and standardized, whereas categorical variables were one-hot encoded before their inclusion in Variant C.

Table S2. Original Blender frame intervals manually selected for the right and left support phases of the five Move AI/Blender participants. The final frame of each interval was treated as inclusive.

Participant	Right support, frames	Left support, frames
Participant 1	357–418	406–463
Participant 2	391–453	438–496
Participant 3	396–451	440–488
Participant 4	477–523	518–560
Participant 5	478–520	513–554

Table S3. Subject-level direct and reconstructed performance of the final LSTM model across the six GroundLink leave-one-subject-out test folds.

Subject	Reconstructed trial	Direct overall RMSE	Direct GRF RMSE	Direct CoP RMSE	Direct contact accuracy	Reconstructed overall RMSE	Reconstructed GRF RMSE	Reconstructed CoP RMSE	Reconstructed contact accuracy
s001	s001_20220513_walk_1	0.0855	0.0476	0.122	0.9393	0.1034	0.0422	0.1551	0.8927
s002	s002_20220520_walk_1	0.084	0.1003	0.0504	0.9664	0.0385	0.0433	0.0298	0.991
s003	s003_20220523_walk_1	0.0803	0.0734	0.0896	0.9529	0.0493	0.0606	0.0237	0.9896
s004	s004_20220524_walk_2	0.0372	0.0411	0.0304	0.9867	0.0353	0.0403	0.0261	0.9884
s005	s005_20220610_walk_1	0.0576	0.0681	0.0366	0.976	0.0367	0.0416	0.0278	0.9884
s006	s006_20220614_walk_1	0.0612	0.0632	0.0581	0.9754	0.0367	0.0428	0.0251	0.9917
Mean	-	0.0676	0.0656	0.0645	0.9661	0.05	0.0451	0.0479	0.9736
